# Pregabalin and α2δ Subunit Modulation: A Systematic Review of Preclinical Evidence for Intraocular Pressure Regulation and Retinal Neuroprotection

**DOI:** 10.3390/jcm15218681

**Published:** 2026-11-06

**Authors:** Gideon Wall, Zirui Zhou, Benton Chuter, Caroline Owens, Noah Emmert, Mohammad Delsoz, Mohamed Moustafa Ibrahim, Monica M. Jablonski

**Affiliations:** 1 College of Medicine, University of Tennessee Health Science Center, Memphis, TN 38163, USA; gwall7@uthsc.edu (G.W.); zzhou24@uthsc.edu (Z.Z.); cowens27@uthsc.edu (C.O.); 2 Department of Ophthalmology, Hamilton Eye Institute, University of Tennessee Health Science Center, Memphis, TN 38163, USA; bchuter@uthsc.edu (B.C.); mdelsoz@uthsc.edu (M.D.); mibrahi2@uthsc.edu (M.M.I.); 3 Department of Computer Science and Software Engineering, Miami University, Oxford, OH 45056, USA; nemmert@uthsc.edu; 4 Department of Pharmaceutics, Faculty of Pharmacy, Mansoura University, Mansoura 35516, Egypt

**Keywords:** glaucoma, intraocular pressure, retinal ganglion cell, pregabalin, gabapentin, α2δ subunit, CACNA2D1, CACNA2D2, voltage-gated calcium channels, excitotoxicity, thrombospondin, neuroprotection, neurodegeneration

## Abstract

**Background:** Glaucoma is a multifactorial optic neuropathy characterized by progressive retinal ganglion cell (RGC) loss, in which both intraocular pressure (IOP)-dependent and -independent mechanisms contribute to disease progression. Gabapentinoids target α2δ subunits of voltage-gated calcium channels (VGCCs), which regulate calcium-dependent processes relevant to both aqueous humor dynamics and retinal neuronal function. This systematic review evaluates heterogeneous evidence relevant to α2δ modulation of IOP regulation, retinal neuroprotection, and associated molecular and synaptic pathways. **Methods:** A systematic literature search was conducted across PubMed, Embase, Scopus, and Cochrane Library databases from inception. Studies investigating gabapentinoids or α2δ subunit modulation with outcomes related to IOP, RGC survival, or relevant molecular signaling were included. Risk of Bias (RoB) was assessed using the OHAT rating tool, and Certainty of Evidence (CoE) was rated on the GRADE four-level scale using initial confidence criteria from the OHAT approach. Due to heterogeneity in study design and outcomes, findings were synthesized narratively, with included studies divided and assessed separately within anterior segment pharmacology, anterior segment genetics, neuroprotection, and mechanistic bodies of evidence. **Results:** Of 1973 unique records screened, 11 studies met inclusion criteria, which were further subdivided into 15 experimental arms according to evidence type. Distinct evidence bodies were anterior segment pharmacology (5 study arms), anterior segment genetics (3 study arms), neuroprotection (4 study arms), and mechanistic (3 study arms). Across mouse and rabbit models of spontaneous ocular hypertension, topical pregabalin (PRG) consistently reduced IOP in a dose-dependent manner, with peak effects generally occurring 1.5–5 h after administration depending on strain and formulation. Plateaus in maximal IOP reduction were observed at 0.9% PRG solution in mice and 0.6–0.7% PRG microemulsion (ME) in rabbits. ME formulations of PRG consistently prolonged duration of action and increased cumulative exposure relative to non-ME PRG formulations. PRG ME achieved maximal IOP reduction comparable to that of 0.005% latanoprost solution while retaining their increased duration of action and cumulative exposure. Repeated once-daily PRG ME dosing maintained IOP-lowering responses over 21- and 60-day treatment periods. Genetic studies separately associated *CACNA2D1*/*Cacna2d1* with IOP-related phenotypes and primary open-angle glaucoma susceptibility, providing target-level evidence. Preclinical posterior segment studies reported retinal structural preservation or improved RGC survival with PRG, while separate studies of GBP-L demonstrated RGC-survival effects associated with K-ATP channel activity rather than established α2δ modulation. Mechanistic studies separately identified thrombospondin-2 (TSP2)-α2δ1 signaling as a mediator of injury-associated synaptic remodeling, with pharmacologic disruption attenuating synaptic protein upregulation and improving axonal integrity. RoB varied across study arms and bodies of evidence. Anterior pharmacology included five Tier II study arms, anterior genetics included two Tier I arms and one Tier II arm, and all neuroprotection and mechanistic arms were Tier II. CoE was rated moderate for both anterior pharmacology and genetics, and low for both neuroprotection and mechanistic evidence. **Conclusions:** The available evidence is predominantly preclinical and heterogeneous. Moderate certainty within the defined anterior segment evidence body reflects consistency of the reported preclinical effect and does not establish clinical efficacy or independent reproducibility, as the topical PRG intervention literature is concentrated within a single investigative group. The neuroprotective and mechanistic findings remain hypothesis-generating and do not establish a unified mechanism or simultaneous IOP-lowering and neuroprotective action. Independent pharmacologic replication, validation in chronic glaucomatous neurodegeneration models, and long-term topical safety evaluation are prerequisites to discussions of clinical relevance.

## 1. Introduction

Glaucoma is a group of optic neuropathies characterized by progressive degeneration of retinal ganglion cells (RGCs) and their axons, ultimately leading to irreversible vision loss [1,2]. Primary open-angle glaucoma (POAG), the most common subtype, is a multifactorial disease in which both intraocular pressure (IOP)-dependent and IOP-independent factors contribute to disease progression [2]. Although IOP reduction remains the only proven disease-modifying therapeutic strategy, a substantial proportion of patients continue to exhibit structural and functional decline despite adequate pressure control [3,4]. Disease progression despite IOP reduction highlights the multifactorial nature of glaucoma and underscores the contribution of additional mechanisms driving RGC degeneration independent of IOP, including neuronal susceptibility, vascular factors, and molecular signaling pathways [5].

Calcium signaling plays distinct regulatory roles in anterior and posterior segment physiology. In the anterior segment, calcium-dependent processes contribute to regulation of AH production and outflow [5,6], while in the posterior segment, calcium homeostasis is essential for synaptic transmission and neuronal survival [7]. Dysregulation of intracellular Ca^2+^, particularly in the setting of excitotoxic stress, has historically been implicated in RGC degeneration [7]. While calcium channel-associated proteins are known to be active in both compartments, it is unknown whether modulation of these proteins could plausibly contribute to each process independently.

**Figure 1:**
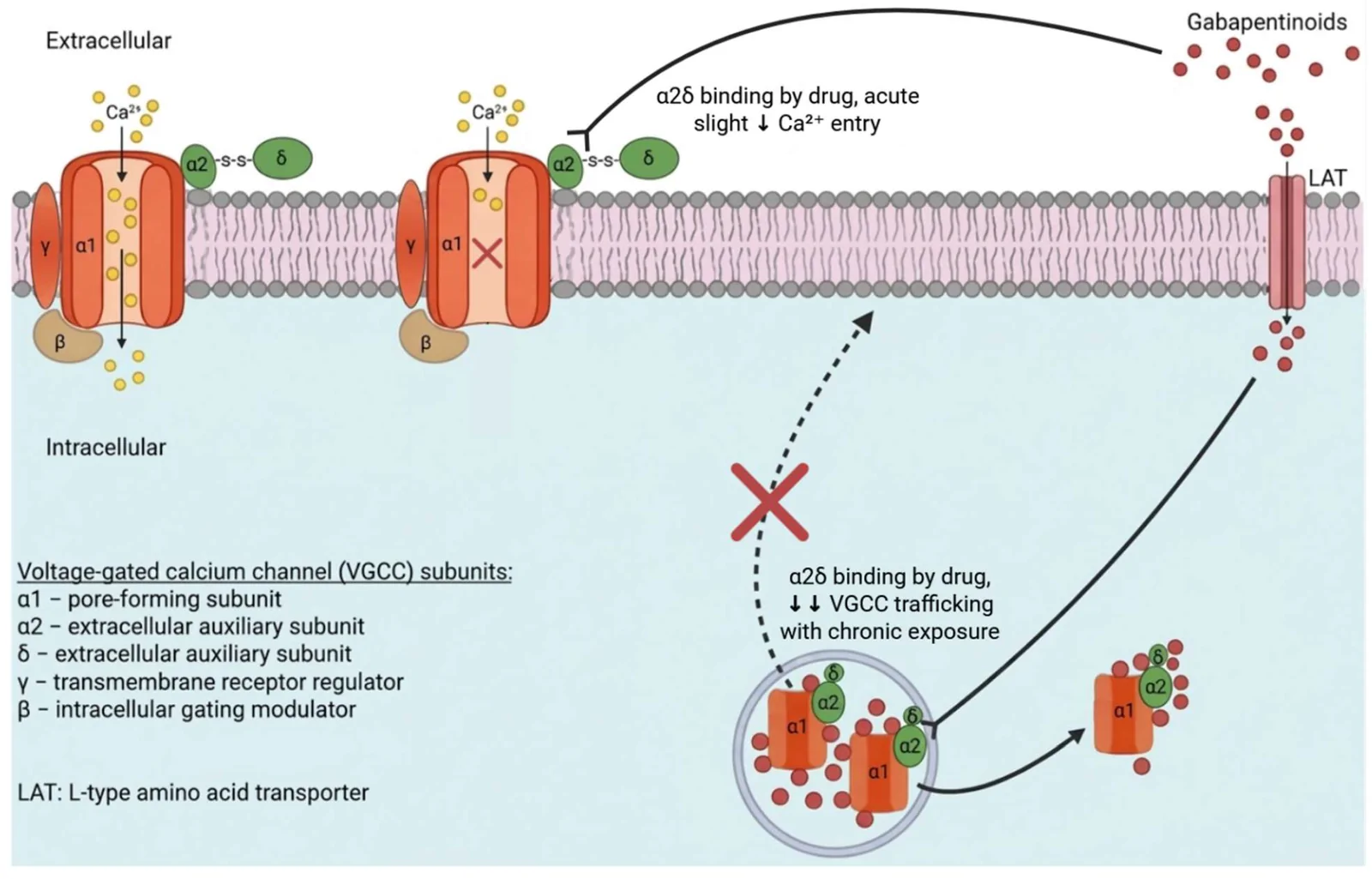
Proposed schematic model based on the integration of preclinical studies of gabapentinoid binding and voltage-gated calcium channel (VGCC) trafficking. Gabapentinoids bind the α2δ (predominantly α2δ1 > α2δ2) auxiliary subunits of VGCCs. While binding occurs extracellularly, intracellular accumulation following L-type amino acid transporter (LAT)-mediated uptake is predicted to contribute to longer-term effects on channel trafficking. Current evidence indicates that reduced Ca^2+^ influx occurs predominantly through impaired α2δ-dependent trafficking and recycling, leading to decreased membrane expression of VGCCs after prolonged gabapentinoid administration.

Gabapentinoids, including gabapentin (GBP) and pregabalin (PRG), are widely used pharmacologic agents that bind the α2δ subunits of voltage-gated calcium channels (VGCCs), modulating calcium channel trafficking and presynaptic neurotransmission [8,9,10,11]. Although originally developed for neurological conditions such as epilepsy and neuropathic pain [12], emerging preclinical evidence suggests that α2δ-dependent signaling pathways may have relevance to ocular physiology. Among calcium channel-associated proteins, the α2δ1 and α2δ2 subunits, encoded by *CACNA2D1* and *CACNA2D2*, respectively, are of particular interest. α2δ subunits are thought to increase the density of VGCCs at the plasma membrane, supporting their theorized role in channel trafficking [8,11]. Binding of gabapentinoids to α2δ-containing VGCCs reduces presynaptic Ca^2+^ influx primarily through impaired channel trafficking and reduced surface expression after prolonged exposure [8,9,10,11]. GBP and PRG are transported into neurons through L-type amino acid transporters (LAT), and LAT-mediated uptake may be necessary for their intracellular access and downstream effects on α2δ-dependent channel trafficking [13]. The theorized mechanisms of action of gabapentinoids in calcium channel signaling are summarized in Figure 1.

In addition to its role in calcium channel trafficking, α2δ1 also functions as a neuronal receptor for astrocyte-secreted thrombospondins (TSP1 and TSP2), which are involved in excitatory synaptogenesis [14]. Experimental models of acute retinal injury have implicated TSP-α2δ1 interactions in the stress responses culminating in retinal synaptic remodeling [15,16]. An association between TSP2 upregulation and injury-related synaptic changes is supported by a localization study that demonstrated increased TSP2 expression and α2δ1-associated synaptic changes following acute elevations in IOP [17]. TSP signaling relevance has also been revealed separately in the anterior segment via its influences on extracellular matrix (ECM) dynamics within the TM, with missense variants in mouse TSP1 impairing AH outflow and raising IOP in vivo [18]. Whether this anterior segment role (mediated by TSP1) and the posterior segment role described above (mediated by TSP2) reflect a shared underlying mechanism has not been directly tested.

Genetic and pharmacologic studies further support a role for α2δ1 in glaucoma-related pathways. Systems genetics analyses have identified *CACNA2D1* as a candidate gene associated with IOP variation in mice and glaucoma susceptibility in humans [19]. Additionally, pharmacologic modulation with PRG microemulsion (ME) eye drops has demonstrated IOP-lowering effects in experimental animal models [20,21]. Together, these separate genetic, pharmacologic, and mechanistic studies support the plausibility that α2δ1 is relevant to multiple ocular processes, but they do not establish that these effects are mechanistically integrated or therapeutically linked. Whether α2δ1 has functionally connected roles across these tissues or instead participates independently in distinct anterior and posterior segment processes has not been established.

In addition to α2δ1, the α2δ2 subunit (encoded by *CACNA2D2*) may play a complementary role in glaucomatous optic neuropathy through regulation of axonal growth and regenerative capacity. In the adult central nervous system (CNS), axonal regeneration is limited partly due to intrinsic gene expression programs that suppress growth and structural plasticity [22]. Transcriptomic and functional studies have identified *CACNA2D2* as a negative regulator of axonal regeneration, with expression inversely correlated with growth competence [23]. In non-ocular CNS injury models, experimental inhibition of α2δ2 (including pharmacologic blockade with gabapentinoids) enhances axonal regeneration and functional recovery following neural injury [24]. Although direct evidence in glaucoma models remains limited, these findings suggest that α2δ2-dependent pathways may influence neuronal vulnerability and regenerative potential in the optic nerve.

The relevance of α2δ signaling to glaucoma is further supported by ocular expression patterns. α2δ1 has been detected in anterior segment structures involved in AH dynamics, including the TM, Schlemm’s canal, ciliary body, and iris, while transcriptomic studies also support broader retinal expression across neuronal populations [19,25,26,27,28,29]. By contrast, α2δ2 appears more restricted to neuronal tissues, including inner retinal cell populations [25,26,27,30,31].

**Figure 2:**
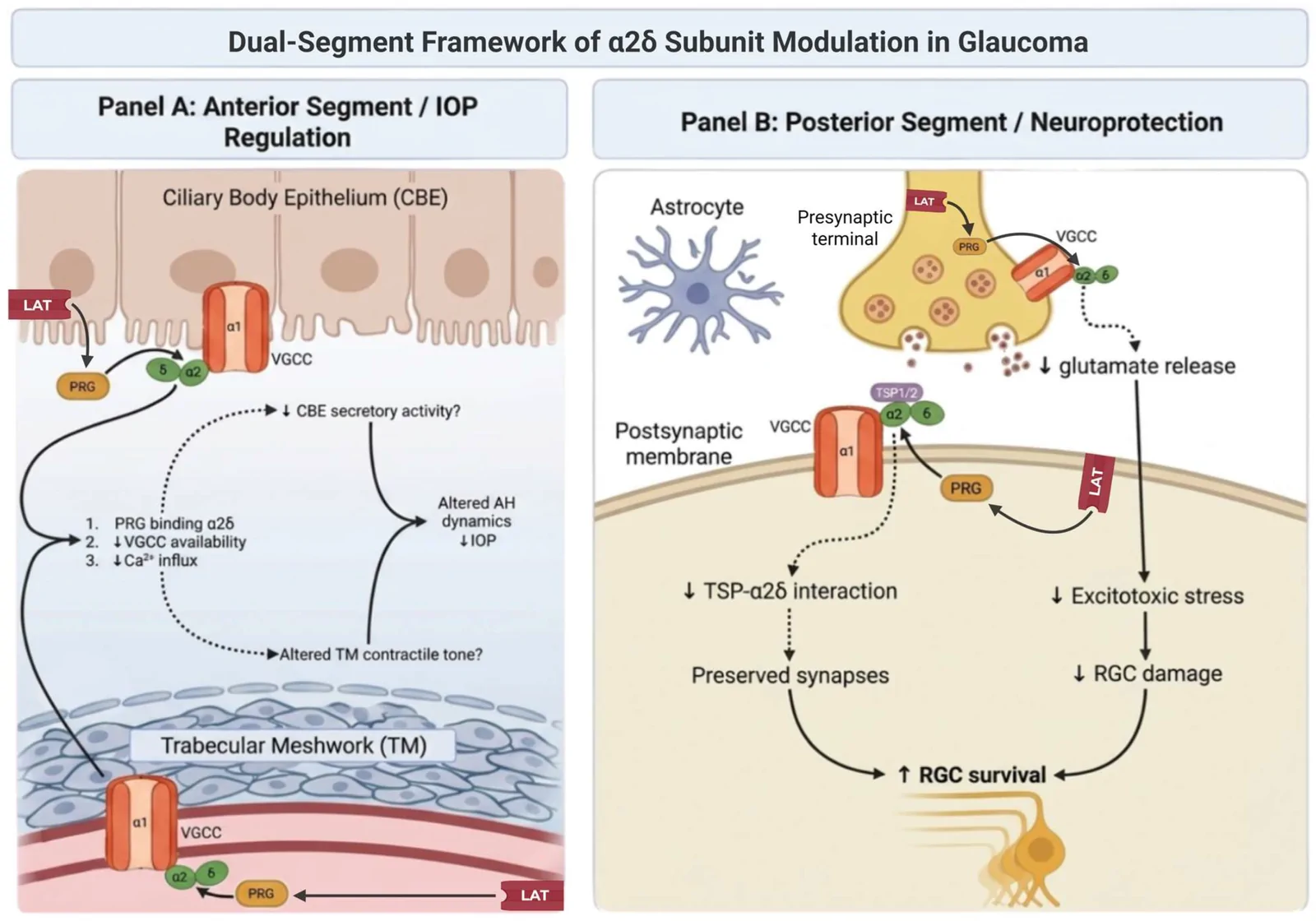
Conceptual, interpretive framework synthesizing preclinical findings across anterior and posterior segment models. (**A**) In the anterior segment, gabapentinoids (represented by PRG for pregabalin) interact with α2δ-containing VGCC complexes in the ciliary body epithelium (CBE) and trabecular meshwork (TM). Mechanistic studies indicate that gabapentinoid binding to α2δ-containing VGCCs reduces presynaptic Ca^2+^ influx primarily through impaired channel trafficking and reduced surface expression, requiring cellular uptake via LAT. Altered aqueous humor (AH) dynamics are our proposed mechanism of reduced intraocular pressure (IOP), though evidence regarding this mechanism is currently very limited (**B**) In the posterior segment, gabapentinoids are proposed to reduce presynaptic Ca^2+^ influx, interfering with glutamate release. Evidence suggests that gabapentinoid binding also reduces thrombospondin (TSP)-α2δ1 signaling, with studies separately indicating that disruptions in TSP-α2δ1 signaling decreases markers of excitotoxic stress and presynaptic modulation, the latter of which has potential maladaptive effects. We hypothesize that disruption of these stress and synaptogenesis pathways by gabapentinoids is correlated with increased retinal ganglion cell (RGC) survival. These two panels summarize experimentally distinct evidence bodies. They do not imply that anterior segment IOP lowering and posterior segment neuroprotection occur through a single mechanism or have been simultaneously demonstrated in a single model or with a single compound.

In summary, glaucoma pathophysiology involves both pressure-dependent stress generated within the anterior segment and early synaptic alterations within the posterior segment that may precede overt RGC loss. Despite growing interest in calcium channel modulation, the existing literature remains heterogeneous, encompassing genetic, molecular, in vitro, in vivo, and clinical studies with varying endpoints and methodologies, and no single study has evaluated α2δ-targeting agents across both compartments within one experimental system. Accordingly, this systematic review evaluates, separately and on their own terms, the evidence for α2δ subunit modulation across three biological areas: (1) anterior segment IOP regulation, (2) RGC survival and neurodegeneration, and (3) underlying molecular and synaptic signaling pathways. Although a single molecular target has been implicated in both the anterior and posterior segments, we consider any proposed “dual-segment” relationship only as a hypothesis-generating framework for future investigation, not as evidence of a validated unified mechanism or therapeutic strategy. A conceptual overview of our hypothetical dual-segment relationship is included in Figure 2.

## 2. Materials and Methods

### 2.1. Study Design

This systematic review was conducted to evaluate the effects of gabapentinoids and α2δ subunit modulation on intraocular pressure (IOP), retinal ganglion cell (RGC) survival, and associated molecular signaling pathways relevant to glaucoma. A review protocol was developed on established systematic review methodology and registered with the International Prospective Register of Systematic Reviews (PROSPERO, record ID: CRD420261384849).

### 2.2. Search Strategy

A comprehensive literature search was performed across PubMed, Embase, Scopus, and Cochrane Library databases from inception to the date of search (22 January 2026). Search terms were designed to capture studies related to gabapentinoids, α2δ subunits (*CACNA2D1*/*CACNA2D2* gene products), TSP signaling, calcium channel biology, and glaucoma-related outcomes, including IOP and retinal neurodegeneration. Search strategies incorporated combinations of controlled vocabulary (e.g., MeSH terms) and free-text keywords. No date restrictions were applied. The search was limited to full-length publications in the English language.

### 2.3. Eligibility Criteria

Studies were included if they met the following criteria:

(a) Investigated gabapentinoids or modulation of α2δ1/2 subunits (*CACNA2D1* or *CACNA2D2* gene products);
(b) Evaluated outcomes related to intraocular pressure, retinal ganglion cell survival, retinal structure/function, or relevant molecular and synaptic signaling pathways;
(c) Utilized human, animal, or in vitro experimental models relevant to ocular physiology or retinal neurobiology.

Studies were excluded if they were:

(a) Review articles, editorials, conference abstracts, or case reports;
(b) Not available as full-length publications;
(c) Not published in English;
(d) Not relevant to ocular or retinal outcomes.

Eligibility criteria were intentionally broad with respect to experimental models and intervention compounds to capture relevant ocular outcomes and mechanistic evidence. Studies of gabapentin derivatives, including GBP-L, were retained for their retinal outcomes without presuming α2δ-mediated activity. The non-ocular hippocampal experiment within Jehle et al. was considered indirect mechanistic evidence accompanying an included retinal neuroprotection study. The resulting heterogeneity in experimental relevance and compound pharmacology is addressed in Section 4.2, Section 4.3, Section 4.4, Section 4.5 and Section 4.6.

### 2.4. Study Selection

**Figure 3:**
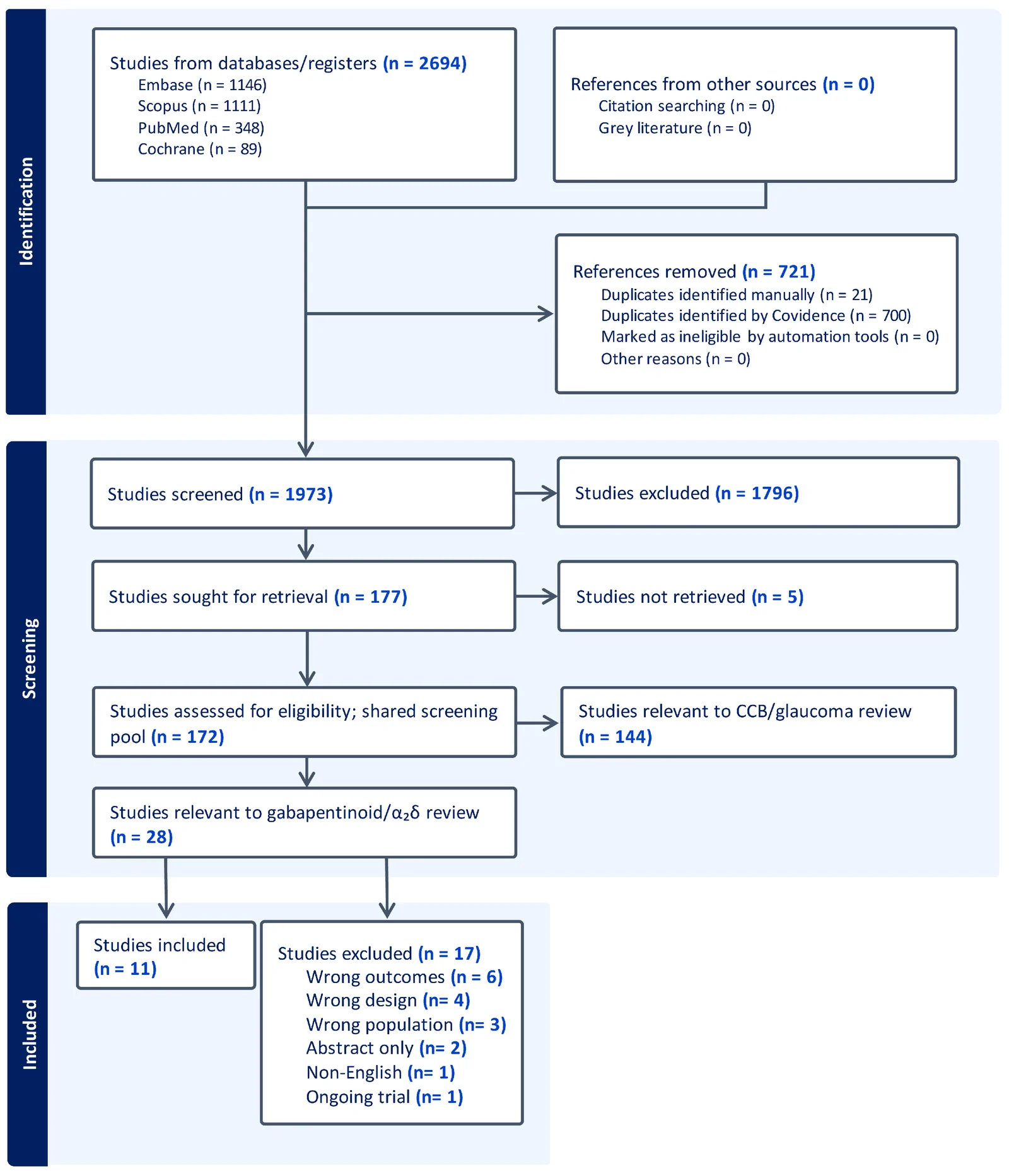
PRISMA flow diagram illustrating the identification, screening, eligibility assessment, and inclusion of studies. Studies were identified through a shared screening process and subsequently allocated to the present review based on predefined eligibility criteria specific to gabapentinoid and α2δ-related outcomes. Studies not meeting inclusion criteria for the present review were allocated to a separate systematic review evaluating calcium channel blockers and glaucoma-related outcomes.

All records identified through database searching were imported into Covidence for screening and deduplication. Three independent reviewers (G.W., C.B., and M.D.) screened titles and abstracts for eligibility. Full-text articles were subsequently assessed for inclusion based on predefined criteria. Disagreements were resolved through discussion and consensus among reviewers. Studies were identified through a shared systematic screening process and subsequently allocated to the present review or to a separate systematic review on calcium channel blockers based on predefined eligibility criteria specific to gabapentinoid and α2δ-related outcomes. Boundaries distinguishing the present review from a parallel CCB review were defined prior to full-text screening and based on numerous differences, including mechanism of action (α2δ subunit targeting vs. conventional calcium channel blockade), study design (preclinical mechanistic, pharmacodynamic, or genetic association studies vs. human observational studies), and outcome types (animal, cell, or genetic locus changes vs. population level statistics). The study selection process is summarized in the PRISMA flow diagram (Figure 3).

### 2.5. Data Extraction

Data extraction was performed independently by two reviewers (Z.Z. and C.O.) using a structured data collection framework. A third reviewer (G.W.) verified extracted data for accuracy and completeness. Extracted variables included the following:

(a) Study characteristics (authors, year, study design);
(b) Experimental model (species, cell type, or population);
(c) Intervention and comparator details;
(d) Outcome measures (e.g., IOP, RGC survival, molecular signaling endpoints);
(e) Quantitative results (including mean values, variability measures, and reported statistical significance);
(f) Source of data (text, table, or figure).

Where appropriate, quantitative values were derived (visual estimation or digitization using WebPlotDigitizer [32]) from graphical data or transformed from reported metrics (e.g., standard error to standard deviation), with assumptions documented.

### 2.6. Data Synthesis

Due to substantial heterogeneity in study design, experimental models, outcome measures, and reporting metrics, quantitative meta-analysis was not performed. Instead, a structured narrative synthesis was conducted. Results were organized into three bodies of evidence:

(1) Anterior segment IOP regulation;
(2) Posterior segment neuroprotection;
(3) Underlying molecular and synaptic pathways.

Findings were synthesized qualitatively and reported separately within each of the three domains, with emphasis on the consistency of effects and the mechanistic plausibility of findings specific to that domain. Findings from separate domains were not treated as interchangeable or mutually validating evidence. To this end, the anterior segment evidence was further divided into pharmacologic and genetic components for CoE assessments. Where appropriate, quantitative outcomes were summarized descriptively to support interpretation.

### 2.7. Risk of Bias and Certainty of Evidence Methods

Risk of Bias (RoB) assessment was conducted independently by two authors (G.W. and Z.Z.), neither of whom held financial interests in the evaluated interventions or in organizations with commercial interests in their development. It should be noted that both RoB and CoE reviewers were UTHSC College of Medicine students also volunteering as researchers with the Jablonski lab in the UTHSC Department of Ophthalmology. No investigator external to the UTHSC research group participated in study screening, data extraction, or RoB/CoE assessments. Discrepancies were resolved through discussion and consensus, and the senior author was excluded from RoB assessment of studies in which they held a disclosed financial or intellectual-property interest. RoB was assessed using the OHAT rating tool [33]. In vivo animal arms were evaluated as experimental animal (EA) studies, the human genetic association arm as a case–control (CaCo) study, and in vitro arms as EA studies, extending the tool to non-whole-organism designs that share the same controlled, randomizable, blindable structure. OHAT bias questions were interpreted according to the bias mechanism relevant to each design. For example, in paired-eye and standardized in vitro experiments, absent randomization, allocation concealment, or personnel blinding was not automatically rated as high risk when the design made appreciable selection or performance bias unlikely. For genetic animal models in which genotype was inherited rather than experimentally assigned, OHAT questions addressing randomized exposure allocation and allocation concealment were considered not applicable. Potential genotype-group imbalance, residual confounding, clustering, or developmental effects were instead assessed under the remaining applicable bias questions.

CoE was assessed using the GRADE (Grading of Recommendations, Assessment, Development and Evaluations) four-level rating scale (high, moderate, low, very low) [34], with initial confidence determined using the OHAT approach, which adapts GRADE principles to controlled non-human experimental designs [35,36]. Study arms with controlled exposure, correct temporal sequence, individual-level outcome data, and an appropriate comparison group began at high initial confidence. CoE was assessed separately for four bodies of evidence: anterior segment pharmacology, anterior segment genetics, neuroprotection, and mechanistic evidence. Confidence was downgraded for serious concerns regarding RoB, indirectness, inconsistency, imprecision, or publication bias. Domain-level RoB downgrading was based on the distribution and consequence of bias across the full body of evidence rather than the presence of isolated probably high-risk ratings in individual studies. A single-level RoB downgrade was applied when methodological limitations were sufficiently prevalent across the contributing evidence to raise meaningful doubt about the results. Potential publication bias was considered separately from internal validity, including the concentration of topical PRG studies within the UTHSC investigative group. To avoid double-counting, downgrades attributable to the same underlying limitation were combined rather than applied separately across multiple domains.

## 3. Results

### 3.1. Study Selection and Study Characteristics

The shared Covidence screening process identified 11 studies meeting inclusion criteria for the present review. These studies comprised anterior segment investigations of IOP regulation, posterior segment studies of retinal neuroprotection, and mechanistic studies evaluating synaptic remodeling or TSP-α2δ signaling. The included evidence base was predominantly preclinical and experimental, consisting of in vivo animal models, in vitro retinal or TM models, and one human genetic association analysis. A summary of study characteristics is provided in Table 1.

Anterior segment studies primarily evaluated *CACNA2D1*/*Cacna2d1* expression and α2δ1 subunit modulation as targets for reducing IOP. These studies included genetic association, pharmacologic intervention, and formulation-based approaches across mice and rabbit models, with Chintalapudi et al. additionally contributing human genomic evidence [19,20,21,29,37]. Posterior neuroprotection studies evaluated retinal structure or RGC survival in diabetic retinal injury, acute retinal ischemia, and isolated RGC culture models [38,39,40,41]. These comprised two studies of PRG, an established α2δ ligand, and two studies of gabapentin-lactam (GBP-L), a GBP derivative for which there is no direct evidence of α2δ-binding affinity. Mechanistic studies examined TSP2-α2δ1 associated signaling in in vitro and in vivo retinal injury systems and ischemia related neurotransmitter release in non-retinal neural tissue [15,16,40]. Because the included studies differed substantially in model systems, interventions, comparators, outcomes, and statistical reporting, meta-analysis was not performed. Findings were therefore synthesized narratively within the three biologically defined areas described in Section 2.6.

### 3.2. Pharmacologic and Genetic Effects on Intraocular Pressure

**Figure 4:**
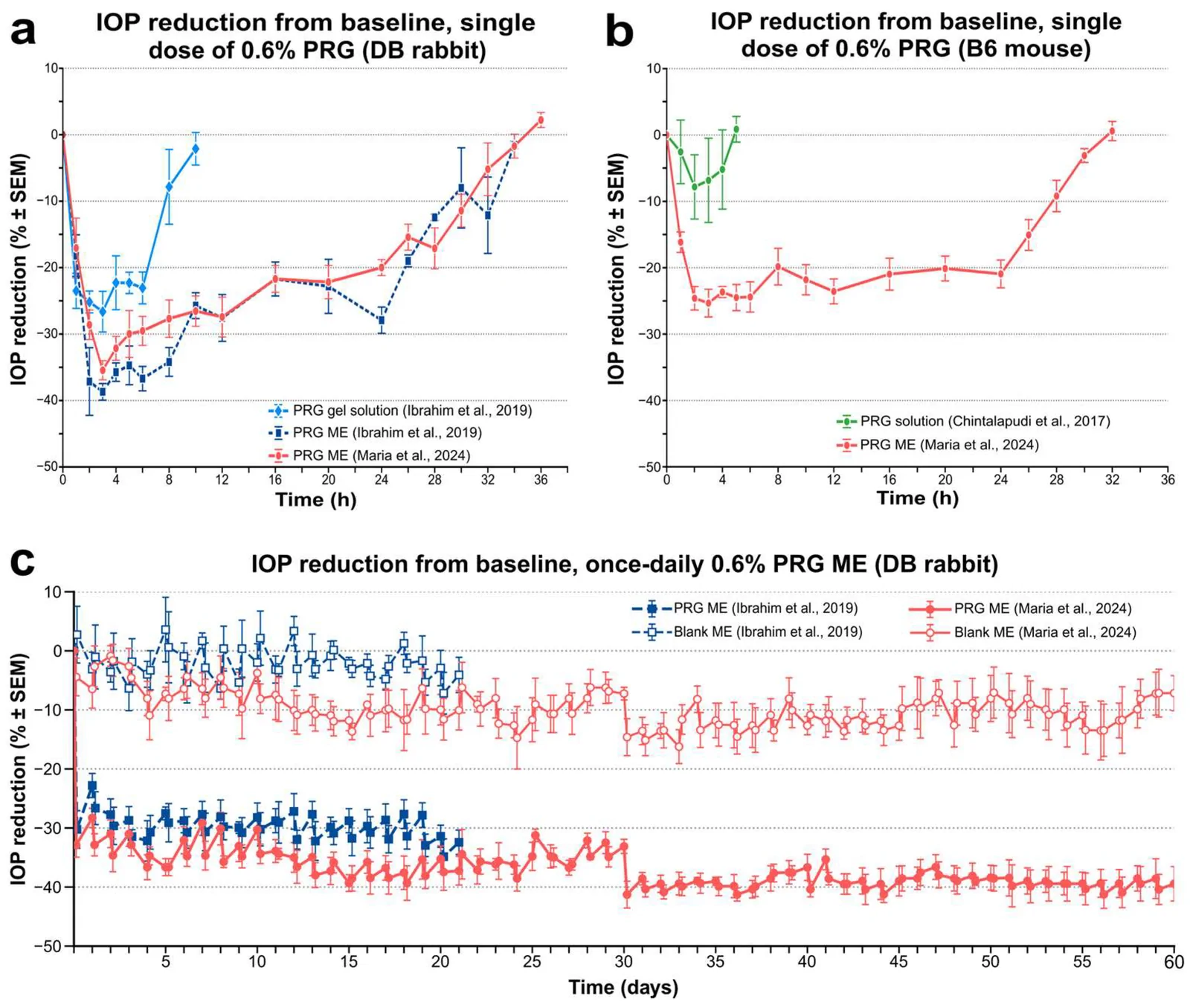
Time-course profiles of IOP reduction after topical application of 0.6% PRG in preclinical models. Profiles are presented descriptively for cross-study visualization. Formal statistical comparison across formulations was not performed. (**a**) In DB rabbits, PRG gel solution and PRG ME produced acute IOP reduction after a single topical dose, with ME formulations achieving more prolonged duration of effect compared with PRG gel solution. Ibrahim et al. evaluated 0.6% PRG gel solution and 0.6% PRG ME in DB rabbits (*n* = 3 per group) [20], whereas Maria et al. evaluated 0.6% PRG ME in DB rabbits (*n* = 5) [21]. (**b**) In B6 mice, 0.6% PRG solution produced a shorter IOP-lowering response, whereas 0.6% PRG ME in B6 mice demonstrated a larger and more sustained reduction over time. Chintalapudi et al. evaluated 0.6% PRG solution in B6 mice (*n* = 6) [19], and Maria et al. evaluated 0.6% PRG ME in B6 mice (*n* = 6) [21]. (**c**) Repeated once-daily administration of 0.6% PRG ME was evaluated in DB rabbits for 21 days by Ibrahim et al. and 60 days by Maria et al. (*n* = 5 in both studies), with the contralateral eye receiving blank ME as the control [20,21]. Both the pre-dose and three hours post-dose IOP measurements from daily dosing cycles are plotted sequentially within the corresponding traces. The PRG ME-treated eyes maintained reduced IOP throughout both repeated-dosing experiments, while blank ME-treated eyes remained closer to baseline. All values are plotted as percent IOP change from baseline over time, with more negative values indicating greater IOP reduction. All data are presented as mean ± SEM. Time-course values were obtained directly from authors, as published graphical data did not report raw hourly numerical values.

Within multiple in vivo anterior segment studies, topical PRG was associated with reductions in IOP in accepted models of spontaneous ocular hypertension, including C57BL/6J (B6) mice, BXD mice, and Dutch belted (DB) rabbits [19,20,21,37]. In these pharmacologic studies, PRG-containing formulations were generally administered to one eye, with the contralateral eye receiving blank vehicle or formulation control. Otherwise, experimental protocols varied by species, strain, formulation, concentration, dose volume, and duration of follow-up, precluding direct pooled comparison. Accordingly, time-course data are presented only descriptively to illustrate pharmacodynamic patterns across studies. 0.6% PRG formulations were represented across all the in vivo topical pharmacology studies and were thus chosen as the treatment condition for these illustrations. Both solution- and ME-based formulations of 0.6% PRG achieved measurable IOP reduction, but the duration of effect differed substantially between studies on solutions versus studies on MEs. In DB rabbits, 0.6% PRG gel solution produced an acute reduction that returned toward baseline within hours [20], whereas 0.6% PRG ME formulations demonstrated more prolonged IOP-lowering effects extending beyond 24 h [20,21]. A similar pattern was observed in mice, particularly the B6 strain, where 0.6% PRG solution [19] managed a shorter IOP reduction response than 0.6% PRG ME [21]. These time-course profiles are summarized below in Figure 4.

**Figure 5:**
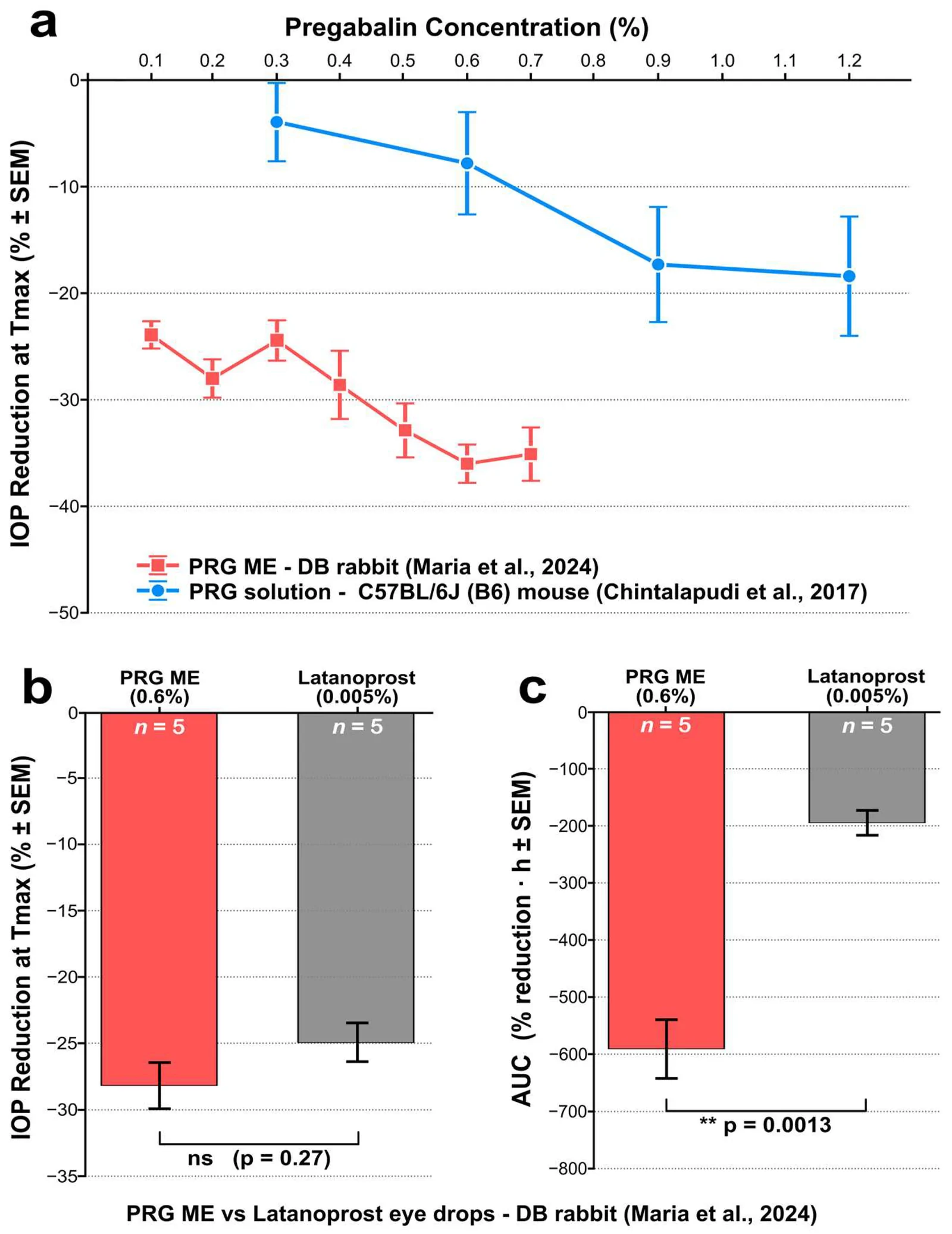
Pharmacodynamic profile of PRG in preclinical models of IOP reduction. Underlying studies differed in species, dosing, and experimental conditions. This figure should be considered a descriptive cross-study visualization of pharmacodynamic parameters. (**a**) Dose–response relationship for PRG administered as topical solution in B6 mice (0.3%, 0.6%, 0.9%, 1.2%) [19] and as ME in DB rabbits (0.1–0.7%) [21], demonstrating increasing IOP reduction at T_max_ with concentration and a plateau in peak effect at ≥0.6% in the Dutch belted (DB) rabbit ME model [21]. Dose-dependent effects were observed in both models (one-way ANOVA: mouse *p* = 0.034, *n* = 6 per group; rabbit *p* = 0.0016, *n* = 5 per group). (**b**) Comparison of peak IOP reduction at T_max_ between PRG ME (0.6%) and latanoprost (0.005%) in the DB rabbit reveals no statistically significant difference in maximal IOP reduction (*p* = 0.27; *n* = 5) [21]. (**c**) Area under the curve (AUC; %·h) comparison between PRG ME (0.6%) and latanoprost (0.005%) demonstrates significantly greater cumulative IOP-lowering exposure for PRG ME (*p* = 0.0013; *n* = 5) [21]. Values are plotted as percent IOP change from baseline over time, with more negative values indicating greater IOP reduction. All data are presented as mean ± SEM.

Dose-ranging experiments established concentration-dependent IOP reduction, with peak effects occurring approximately 1.5–5 h after administration depending on strain and formulation [19,21]. Dose-dependent effects were observed in both mouse and rabbit models, with significant overall differences in IOP reduction across concentrations (ANOVA: mouse *p* = 0.034 [19]; rabbit *p* = 0.0016 [21]). Formulation-dependent effects in both species were pronounced when pharmacodynamic parameters beyond peak reduction were considered. ME-based formulations of PRG produced substantial prolongation of effect duration compared with non-ME formulations in mice and DB rabbits [20,21]. Consistent with this interpretation, Maria et al. demonstrated that increasing PRG concentration in the ME primarily increased T_end_ and AUC while peak IOP reduction reached a plateau [21]. Comparative benchmarking against latanoprost revealed similar pharmacologic differences. While peak IOP reduction did not differ significantly between PRG ME and latanoprost (*p* = 0.27), PRG ME achieved significantly greater cumulative exposure (AUC; *p* = 0.0013), consistent with a prolonged pharmacodynamic profile rather than increased peak potency [21]. These dose–response, formulation-dependent, and comparative effects are summarized below in Figure 5.

An additional pharmacologic validation study performed by Li et al. resulted in PRG producing greater IOP reduction than comparator agents under similar conditions. In DB rabbits, PRG achieved larger peak reductions and greater overall exposure compared with acivicin and zoledronic acid [37].

Ibrahim et al. and Maria et al. additionally evaluated ocular PRG distribution following topical ME administration in DB rabbits [20,21]. Ibrahim et al. demonstrated measurable PRG concentrations in posterior segment tissues, including the retina and vitreous humor, following 21 days of once-daily dosing [20]. Maria et al. subsequently demonstrated PRG exposure in both anterior and posterior ocular tissues, including the retina, vitreous humor, and optic nerve, over a 1–6 h sampling period following a single topical dose. PRG ME produced greater tissue exposure than aqueous PRG, including significantly increased retinal, vitreous, and optic nerve bioavailability [21]. Separate measurements following 60 days of once-daily administration demonstrated PRG accumulation in retinal and optic nerve tissues. These experiments established posterior segment drug exposure with topical ME formulations but did not evaluate retinal neuroprotective efficacy [20,21].

Genetic evidence supported a role for *Cacna2d1* gene products in IOP regulation. In mouse strain comparisons, the magnitude of IOP reduction following PRG administration varied across genetic backgrounds, with strains possessing higher relative *Cacna2d1* expression (e.g., B6, BXD44, BXD14 [19,21]; *b* allele) demonstrating greater percent IOP reduction compared with strains with lower expression (e.g., DBA/2J (D2), BXD48 [19]; *d* allele). Complementary evidence of *Cacna2d1* null mice exhibiting lower baseline IOP compared with wild-type controls was also revealed [29]. In the human GWAS component of Chintalapudi et al., *CACNA2D1* variation was associated with POAG susceptibility in the NEIGHBORHOOD cohort [19].

### 3.3. Effects on Retinal Ganglion Cell Survival and Neurodegeneration

**Figure 6:**
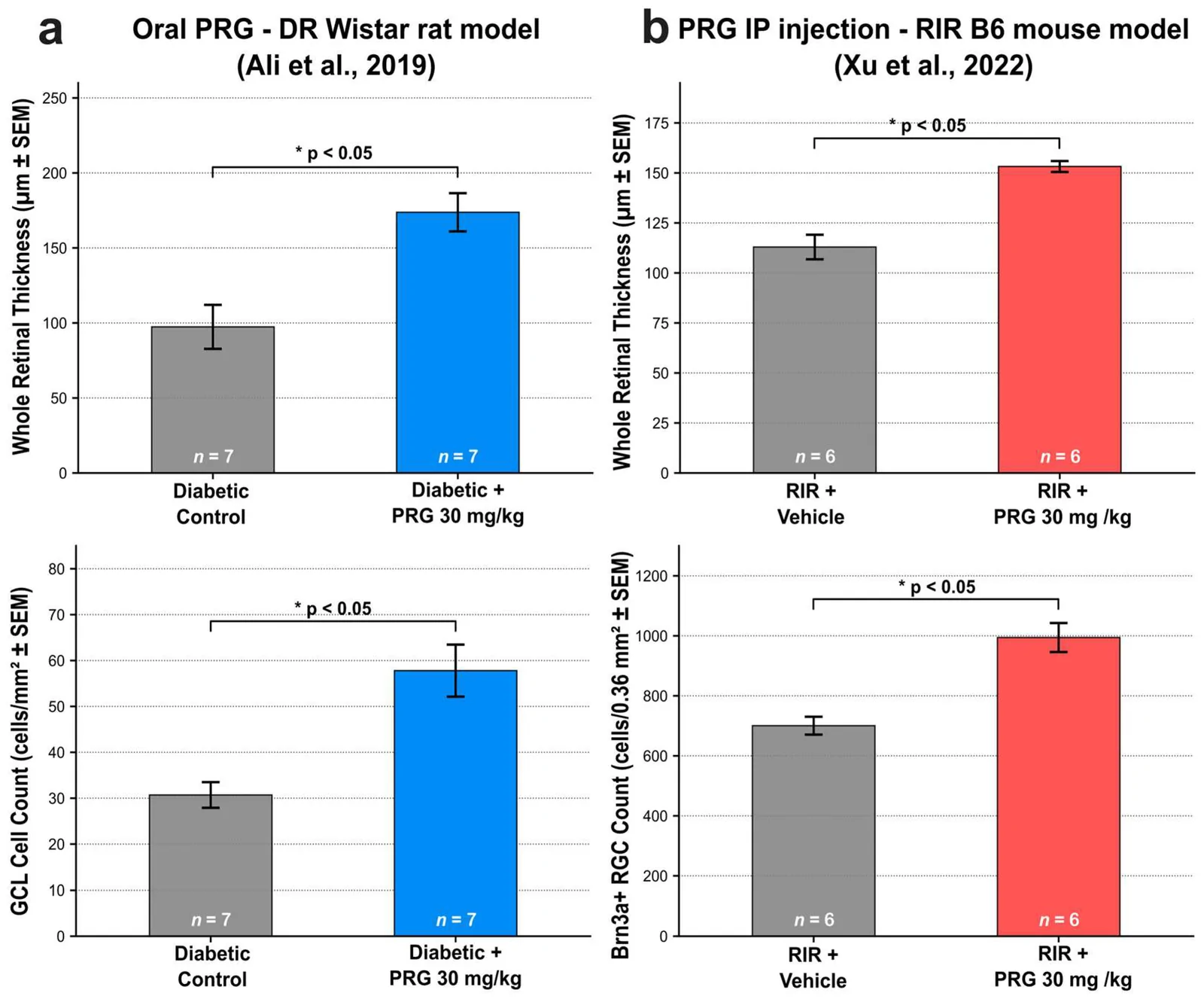
Effects of PRG on retinal structure and RGC survival across experimental injury models. Separate panels present data from independent studies using two different injury paradigms. Data are shown together only as a descriptive visual summary of systemic PRG neuroprotective effects—no direct statistical comparison between studies was performed. (**a**) In a streptozotocin-induced DR Wistar rat model, systemic PRG (30 mg/kg oral) was associated with increased whole retinal thickness (top) and higher GCL cell counts (bottom) compared with untreated diabetic rat controls [38]. Statistical comparisons reflect between-group differences within the diabetic model (*p* < 0.05; *n* = 7 per group). (**b**) In an RIR injury B6 mouse model, PRG (30 mg/kg IP) increased whole retinal thickness (top) and Brn3a-positive RGC counts (bottom) relative to vehicle-treated controls [39]. Statistical comparisons reflect between-group differences within the RIR model (*p* < 0.05; *n* = 6 per group for histologic analysis). All data are presented as mean ± SEM. Quantitative values were digitized from published graphical data in the original studies where raw numerical data were not reported.

In both retinal ischemia–reperfusion (RIR) and diabetic retinopathy (DR) rodent models of acute retinal injury, systemic PRG administration was associated with increased retinal thickness and preservation of RGC-related cellular metrics compared with untreated controls (*p* < 0.05 for all comparisons) [38,39]. Ali et al. evaluated oral PRG administration (3, 10, or 30 mg/kg daily for five weeks) in a streptozotocin-induced diabetic rat model [38]. Xu et al. evaluated intraperitoneal PRG pretreatment (15, 30, or 60 mg/kg) in a mouse RIR model, with additional experiments assessing PRG-mediated protection in primary RGC cultures exposed to oxygen-glucose deprivation/reoxygenation [39]. Heterogeneity in experimental protocols precluded direct pooled comparison. Structural and cellular outcomes were summarized below in Figure 6 only for descriptive visual comparison.

Additional neuroprotection evidence was provided by Jehle et al. and Pielen et al. papers, both of which evaluated GBP-L (a cyclic amide derivative of GBP). In an in vivo rat model of pressure-induced retinal ischemia, GBP-L increased RGC survival relative to vehicle, whereas GBP did not [40]. In purified rat RGC cultures, GBP-L increased cell survival with max effect plateau at intermediate concentrations (32 μM), while GBP was ineffective at the same concentration [41]. GBP-L’s protective effects were abolished by pharmacologic K-ATP channel inhibitors [41]. While this evaluation of K-ATP channel blockade as a potential mediator of this survival effect falls more within the mechanistic domain of evidence, the primary contributions of these two papers were classified as neuroprotective because the principal outcomes were related to RGC survival. The included papers presented no evidence about the α2δ-binding affinity of GBP-L.

### 3.4. TSP2-α2δ Signaling and Ischemia-Related Neurotransmitter Release

**Figure 7:**
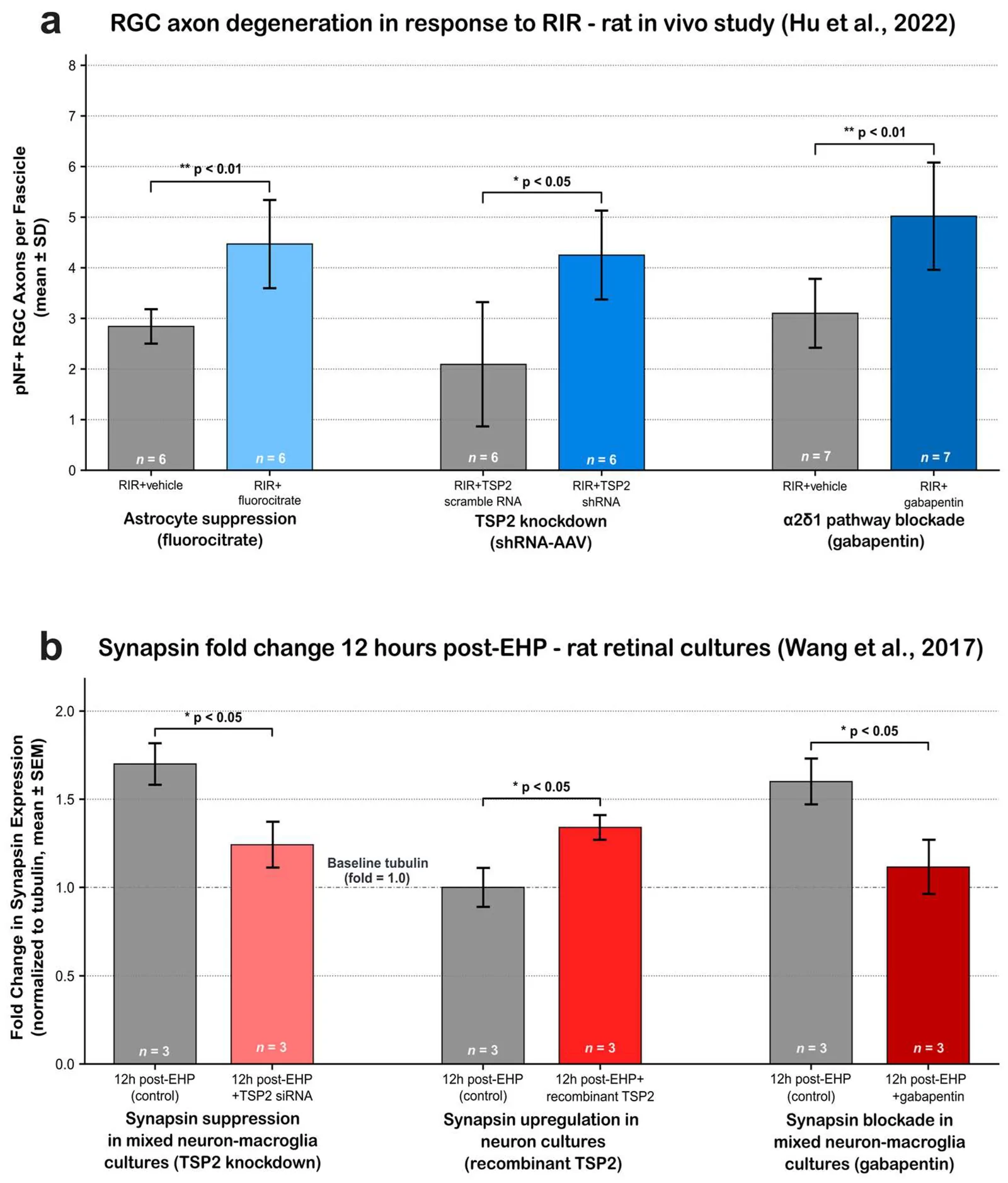
TSP2-α2δ1 associated changes in RGC axonal integrity and synaptic protein expression in rat retinal injury models. Separate panels present data from independent studies using two different injury model systems. Data are shown together only as a descriptive visual summary of TSP2-α2δ1 pathway evidence. No direct statistical comparison between studies was made. (**a**) In an in vivo rat RIR injury model, pharmacologic suppression of glial activity with fluorocitrate, TSP2 knockdown via AAV2-delivered shRNA, and GBP treatment were each associated with increased pNF-H+ RGC axons per fascicle. Fluorocitrate and GBP treatments were matched with phosphate buffer vehicle, while the TSP2 knockdown treatment was matched with scramble RNA-AAV2 to support a TSP2-specific effect. Statistical comparisons reflect between-group differences within each intervention (fluorocitrate: *p* < 0.01; *n* = 6. TSP2 shRNA: *p* < 0.05; *n* = 6. GBP: *p* < 0.01; *n* = 7) [16]. (**b**) Rat retinal cultures were exposed to elevated hydrostatic pressure (EHP) for 2 h, with experiments performed 12 h post-EHP. TSP2 siRNA knockdown and GBP treatment (in mixed neuron-macroglia cultures) were associated with reduced synapsin expression compared with EHP alone, whereas recombinant TSP2 treatment (in neuron cultures) increased synapsin expression relative to vehicle. Statistical comparisons reflect between-group differences within each experimental condition (*p* < 0.05; *n* = 3 per group) [15]. Synapsin expression is reported as fold change normalized to baseline, defined as 1.0. Quantitative values were digitized from published graphical data in the original studies where raw numerical data were not reported.

Mechanistic studies evaluated α2δ-associated signaling in retinal injury and pressure-stress models. In an in vitro retinal neuron-macroglia co-culture system exposed to acutely elevated hydrostatic pressure (EHP), macroglia-derived TSP2 was associated with increased expression of presynaptic proteins in retinal neurons, including synaptophysin and related synaptic markers [15]. Knockdown of TSP2 with siRNA reduced EHP-induced synapsin 1 expression, whereas exogenous recombinant TSP2 increased synapsin 1 expression. Like TSP2 knockdown, pharmacologic treatment with GBP attenuated pressure-induced increases in synapsin 1 expression [15]. Hu et al. extended this pathway experimentally to an in vivo rat RIR model. Pharmacologic suppression of glial activity, AAV2-mediated TSP2 knockdown, and GBP treatment were associated with attenuation of injury-related presynaptic protein changes, improved RGC axonal organization, and improved visual-function measures [16]. A distinct mechanistic arm examined ischemia-related neurotransmitter release in rat hippocampal slices. GBP-L reduced oxygen-glucose deprivation-induced [^3^H]glutamate release, an effect abolished by the KATP-channel blocker glibenclamide. GBP also reduced glutamate release under the same conditions, although to a lesser extent [40]. Because this specific experiment used non-retinal tissue and addressed neurotransmitter-release pharmacology rather than neuronal survival outcomes, it was considered mechanistic evidence separately from the in vivo neuroprotective arm described in Section 3.3. These findings are summarized descriptively in Figure 7 below.

### 3.5. Risk of Bias and Certainty of Evidence Results

**Figure 8:**
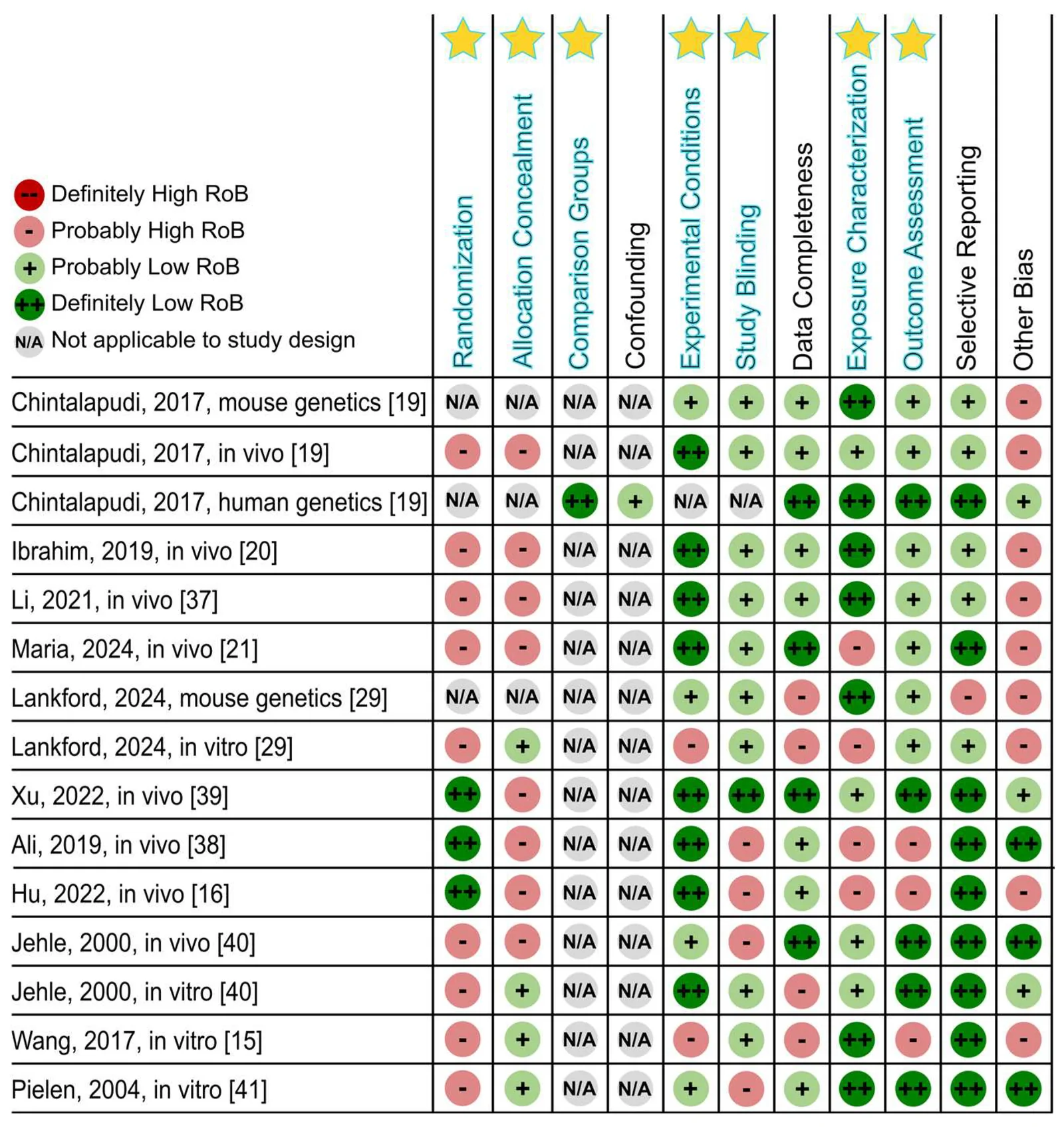
Assessment of the internal validity of reviewed evidence using the OHAT Risk of Bias (RoB) rating tool. Cell color and symbol indicate the judgment for each OHAT question, as defined in the figure legend. Starred column headings denote OHAT key elements used in study-tier classification. Full study-arm-level assessments, tier assignments, and rationale are provided in Table S6.

Full study-arm-level judgments, rationale, and tier assignments are provided in Table S6. RoB results are summarized below in Figure 8.

RoB profiles varied across study arms and experimental designs. All five anterior segment pharmacology arms were classified as Tier II, while the anterior segment genetic evidence comprised two Tier I arms and one Tier II arm. In paired-eye pharmacologic experiments, absence of formal randomization and allocation concealment were recurring concerns, though the practical ability of these deficits to significantly bias results is relatively low. Lack of personnel blinding was not considered intrinsically high risk when the within-animal design made appreciable selection or performance bias unlikely. Randomization and allocation-concealment domains were considered not applicable in inherited genetic models. All four neuroprotection arms (Ali et al., Xu et al., Jehle et al. in vivo, and Pielen et al.) and all three mechanistic arms (Wang et al., Hu et al., and Jehle et al. in vitro) were classified as Tier II, reflecting differing study-specific limitations rather than a single recurrent source of bias. No study arm was classified as Tier III.

Tier assignments from the RoB assessments were then grouped by evidence type (anterior segment pharmacology, anterior segment genetics, neuroprotection, and mechanistic) and applied to OHAT’s guidance on confidence rating across a body of evidence [36]. CoE was assessed separately for each of these evidence-type groups (Table S7). Anterior segment pharmacology was rated as moderate certainty after downgrades for RoB, indirectness, and risk of publication bias (all studies originating from a single investigator group) were partially offset by upgrades for cross-species consistency of PRG-produced IOP reduction and the monotonic pattern of the PRG dose–response gradient. Anterior segment genetic evidence was rated as moderate certainty after downgrades for indirectness and publication bias and an upgrade for consistency across genetic analyses. Neuroprotection and mechanistic evidence were each rated as low certainty after downgrades for RoB and indirectness, with no applicable upgrades. Regarding the neuroprotection evidence body, it should be noted that the two GBP-L studies supported neuroprotection outcomes but did not provide direct evidence of α2δ mediation of these outcomes, which was factored into downgrades for indirectness.

## 4. Discussion

### 4.1. Principal Findings and Overall Interpretation

This systematic review summarizes predominantly preclinical evidence on α2δ subunit modulation in the context of IOP regulation, RGC survival, and synaptic remodeling relevant to glaucoma. Consistent with the heterogeneity of the evidence included, findings are considered separately by evidence type and by disease-model relevance rather than treated as a single translational claim.

Across anterior segment models of spontaneous ocular hypertension, pharmacologic modulation with PRG was consistently associated with IOP reduction. Despite the heterogeneity of this preclinical body of evidence, there is sufficient overlap in findings to postulate a pharmacodynamic profile of PRG that includes an early peak effect, a plateau in maximal IOP reduction at moderate-high concentrations of PRG depending on formulation, as well as substantial formulation-dependent prolongation of duration and exposure [19,20,21]. This pharmacologic evidence achieved moderate certainty within the defined preclinical evidence base, but all contributing intervention studies originated from a single investigative group as previously disclosed in Section 3.5 and addressed further in Section 4.6. Accordingly, internal consistency across experiments should not be equated with independent reproducibility.

Genetic evidence across three study arms from two papers separately associated *CACNA2D1*/*Cacna2d1* with IOP-related phenotypes in mice and POAG susceptibility in humans [19,29]. These genetic findings were assessed separately from the pharmacologic evidence and support target-level plausibility rather than independent replication of topical PRG efficacy.

In the posterior segment, PRG was associated with preservation of retinal structure or RGC survival-related outcomes in diabetic retinal injury and acute retinal ischemia models [38,39]. Separate studies of GBP-L reported improved RGC survival in acute retinal ischemia models and purified RGC cultures, although its α2δ-binding affinity remains unestablished in both the included studies and the wider literature [40,41]. These findings provide low-certainty evidence of neuroprotective effects within the specific experimental systems studied due to study-level RoB limitations and substantial indirectness to chronic glaucomatous neurodegeneration. Furthermore, they do not establish a common α2δ-dependent mechanism.

Mechanistic studies separately implicated TSP2-α2δ1 signaling in retinal synaptic and neuritic responses to acute stress and identified modulation of ischemia-related glutamate release in non-retinal neural tissue [15,16,40]. These findings are consistent with broader evidence identifying α2δ1 as a neuronal receptor for thrombospondins involved in excitatory synaptogenesis [14,42,43]. This mechanistic evidence was likewise rated as low certainty and should not be interpreted as demonstrating a single pathway linking anterior segment IOP effects to posterior segment neuroprotection.

Together, the included evidence identifies several experimentally distinct α2δ-associated phenomena relevant to ocular physiology and retinal injury. Whether these phenomena are functionally connected within chronic glaucoma remains untested.

### 4.2. α2δ1 and IOP Regulation in the Anterior Segment

The anterior segment evidence implicates the *CACNA2D1*/*Cacna2d1* gene and its α2δ1 protein product in IOP-related biology through distinct genetic and pharmacologic findings that differ in design and outcome type. These two areas of anterior segment evidence are therefore considered separately below.

Genetic evidence implicates *CACNA2D1*/*Cacna2d1* in IOP regulation through three separate study arms. First, an external human GWAS consortium linked *CACNA2D1* variation to POAG susceptibility across 3853 cases and 33,480 controls from the NEIGHBORHOOD consortium [19]. Second, mouse systems genetics revealed that *Cacna2d1* expression varied across BXD, B6, and D2 strains in a manner corresponding to the magnitude of pharmacologic IOP response [19]. Third, *Cacna2d1* knockout mice possessed lower baseline IOP than wild-type controls [29]. While the human GWAS component draws on an external consortium cohort, all associations identified by these genetic studies come from the same investigative group responsible for the pharmacologic findings discussed below. These findings provide moderate certainty genetic evidence relevant to α2δ1 target plausibility.

Pharmacologic evidence, entirely from the UTHSC investigative group, demonstrated consistent topical PRG-induced reductions in IOP in mouse and DB rabbit models [19,20,21,37]. The pharmacodynamic profile observed suggests that α2δ1-mediated IOP modulation displays a plateau in response: peak efficacy plateaued at moderate-high concentrations, with formulation strategies primarily extending duration of action and cumulative exposure rather than increasing maximal IOP reduction [19,20,21]. Comparisons with latanoprost further support this distinction, as PRG ME achieved similar peak IOP reduction but longer duration and greater AUC in the studied DB rabbit model [21]. Repeated once-daily dosing also maintained the IOP-lowering response over chronic dosing periods of 21 days in one study and 60 days in a second, with no reported attenuation of effect over the treatment periods [20,21]. A recent publication from the same UTHSC research group reported similar absence of tachyphylaxis during 30 days of once daily dosing with a revised PRG enhanced-delivery formulation (PRG-EDF) in DB rabbits, along with IOP reduction in a cohort of seven baboons using the same intervention [44]. The same study also benchmarked PRG-EDF against timolol, latanoprost, and latanoprostene bunod in DB rabbits, reporting a greater AUC with PRG-EDF administration in comparison to all three commercial agents. These data were not incorporated into the formal evidence synthesis, RoB assessment, or CoE assessment of this review, as the publication appeared after completion of our systematic literature search.

The cellular basis for this IOP-lowering effect remains unresolved. This uncertainty is consistent with broader α2δ biology, as the downstream effects of gabapentinoid binding may depend on cellular context and disease-associated changes in α2δ expression or channel organization [11]. In addition, the requirement for LAT in cellular uptake of gabapentinoids suggests that pharmacokinetic factors, including tissue penetration and intracellular accumulation, may influence the extent of α2δ-mediated modulation in ocular tissues [13]. AH homeostasis depends on ciliary body production and TM-mediated outflow, both of which are regulated by ion transport, cytoskeletal organization, and calcium-sensitive signaling [5,6]. Although α2δ1 has been localized to anterior segment tissues relevant to AH dynamics, in vitro testing with PRG by the same investigative group responsible for the pharmacologic findings above did not produce overt short-term (≤24 h) changes in human TM cell morphology, actin organization, focal adhesions, or contractility-associated signaling [29]. These findings do not support a primary cytoskeletal or Rho kinase-like mechanism as the primary explanation for the observed acute IOP effect, at least in the short-term exposure conditions tested. Overall, the anterior segment pharmacologic findings provide moderate certainty evidence regarding α2δ1 targeting as a means to lower IOP.

TSP-associated pathways may represent an additional interface linking α2δ1 to anterior segment physiology, although current evidence is limited and indirect at that. TSP1 missense variants have been linked with impaired AH outflow, producing TM ECM aggregation in congenital glaucoma models [18]. The following section considers α2δ1-TSP2 interactions in the posterior segment, where this pathway and the involvement of α2δ1 has been examined more directly in retinal injury and synaptic-remodeling models.

### 4.3. Retinal Neuroprotection and Injury Models

The posterior segment findings indicate that gabapentinoid compounds are associated with preservation of retinal structure and RGC survival across multiple experimental injury paradigms. However, the magnitude and consistency of these effects vary by model and compound. Unlike the anterior segment pharmacology discussed in Section 4.2, these findings originate from independent research groups without disclosed institutional or financial interest in the compounds evaluated. Systemic PRG demonstrated protective effects in both RIR and DR rodent injury models, with preservation of retinal thickness, maintenance of GCL and RGC cellularity, and reductions in injury-associated biochemical markers [38,39]. This structural and molecular evidence aligns with established mechanisms of retinal injury involving excitotoxic signaling, metabolic stress, and apoptosis, which have been implicated in RGC degeneration in glaucomatous and non-glaucomatous models [7]. It should be emphasized here that the neuroprotective effects of PRG were demonstrated following systemic administration [38,39], whereas these effects have not been established following topical dosing interventions as in the anterior segment studies of this review (though we do have evidence of posterior segment drug exposure with topical PRG ME [21]).

The GBP-L studies warrant separate consideration regarding the effects of gabapentinoids on the retina. Although GBP-L is structurally derived from GBP, its binding affinity for α2δ subunits has not been directly evaluated. Structural characterization of the GBP-α2δ1 binding pocket identifies interactions involving the free amino and carboxylate groups of GBP, which are altered by lactamization [45]. These differences provide a structural basis for questioning GBP-L binding at the canonical α2δ1 site, although its binding affinity has not been directly evaluated. In both acute retinal ischemia and purified RGC culture, GBP-L increased RGC survival while GBP failed to consistently achieve similar neuroprotection under the experimental conditions evaluated [40,41]. GBP-L’s neuroprotection effects were instead proposed to be related to K-ATP channel activity, particularly in the purified RGC culture model. These findings therefore cannot independently establish α2δ mediation of retinal protection by GBP or GBP-L.

These findings do not represent a common chronic glaucoma model. As stated above, Pielen et al. implicated KATP-channel activity in the GBP-L survival effect while Xu et al. associated PRG neuroprotection with Akt/GSK3β/β-catenin signaling and changes in apoptosis, oxidative stress, and mitochondrial injury [39,41]. Accordingly, the observed survival effects may reflect compound- and model-specific mechanisms rather than uniform gabapentinoid neuroprotective action. The overall certainty of this evidence was low, reflecting both study-level RoB concerns and indirectness. The included models represent diabetic retinal injury, acute retinal ischemia/reperfusion, or isolated RGC culture rather than the prolonged axonal and somatic degeneration characteristic of chronic glaucoma. These findings therefore identify neuroprotection as a hypothesis for further testing rather than establishing efficacy in glaucomatous neurodegeneration.

### 4.4. Thrombospondin-α2δ Signaling and Ischemia-Related Neurotransmitter Release

The mechanistic studies included in this review suggest that TSP-α2δ1 signaling is relevant to retinal injury-associated synaptic alterations. In experimental systems, increased presynaptic proteins including synapsin and synaptophysin were reported following experimental pressure or ischemic injury, with attenuation of these changes following TSP2 knockdown or GBP treatment [15,16]. However, whether increased presynaptic protein expression reflects compensatory remodeling rather than an intrinsically harmful response is not established by these experiments. The observed reduction in protein expression with TSP2-related interventions does not independently establish neuroprotection either. Reporting of improvements in RGC axonal organization and visual-function measures by Hu et al. does provide complementary structural and functional evidence, however. Although retinal TSP2 knockdown was demonstrated following AAV2-mediated shRNA delivery, the vector’s promoter, pseudotype, and cellular specificity were not fully characterized. GFP/GFAP colocalization supported transduction of GFAP-positive glia but did not exclude reactive Müller cells or demonstrate selective astrocyte targeting [16]. Thus, these studies support involvement of glial TSP2-α2δ1 signaling in acute retinal injury, but do not establish an astrocyte-specific mechanism or a definitive contribution to chronic glaucomatous neurodegeneration.

A separate mechanistic experiment revealed that GBP-L and GBP reduced oxygen and glucose deprivation-induced glutamate release in rat hippocampal slices, with the GBP-L effect abolished by KATP-channel blockade [40]. Because this experiment used non-retinal neural tissue and a different pharmacologic mechanism, it should be considered mechanistically informative rather than confirmatory evidence for the retinal TSP2-α2δ1 pathway.

Prior literature provides some context for the role of α2δ1 in neuronal regulation, as astrocyte-derived TSPs promote excitatory synapse formation [43], with α2δ1 subsequently identified as the neuronal receptor mediating this effect [14], and with downstream signaling pathways implicated in synaptic stabilization and structural remodeling [42]. These processes are essential for normal synaptic development but may also be re-engaged or dysregulated under conditions of injury or stress. Early retinal synaptic and neuritic alterations occur prior to overt RGC loss in models of elevated IOP, indicating that circuit-level dysfunction is an early component of disease progression [46,47]. Within this context, TSP-α2δ1 signaling may contribute to stress-associated synaptic remodeling. However, the directionality and functional consequences of these changes remain incompletely defined.

The three mechanistic study arms included in the CoE assessment differ in tissue, injury paradigm, intervention, and endpoint and were therefore considered complementary rather than mutually validating. Their overall certainty was low because of RoB limitations and indirectness to chronic glaucoma.

### 4.5. Review Implications and Future Investigative Considerations

The findings summarized in this review are best interpreted as hypothesis-generating. Apart from one human genetic association study, the evidence is preclinical and heterogeneous, and no included study directly evaluated α2δ-targeting therapy in a chronic glaucoma model. The four evidence bodies also differ materially in intervention, tissue, species, experimental paradigm, and outcome and should not be interpreted as mutually validating components of a unified therapeutic mechanism.

Anterior segment pharmacology evidence included topical PRG demonstrating consistent dose-responsive IOP reduction and formulation-dependent prolongation of effect, including maintained efficacy during repeated once-daily dosing with ME formulations in rabbit models [20,21]. The corresponding moderate CoE rating reflects confidence in the reported effect within this preclinical evidence base, not clinical efficacy. All topical PRG intervention studies originated from the same investigative group, which was incorporated as a publication-bias concern. The separately assessed genetic evidence achieved moderate certainty and provides target-level biological context.

A recent publication by Maria et al. reported sustained IOP reduction in Dutch belted rabbits and a small baboon cohort with topical PRG-EDF, alongside exploratory systemic exposure and comparative pharmacodynamic assessments [44]. The study was published after the systematic search cutoff and was therefore not included in formal analyses. Although these reported findings extend the preclinical investigation of topical PRG and appear consistent with the sustained IOP-lowering responses of the studies included in this review, the study originated from the same UTHSC investigative group and thus does not address the need for independent pharmacologic replication.

Neuroprotective and mechanistic evidence bodies were each assessed as low certainty because of RoB and indirectness. The relevant studies used acute retinal ischemia, diabetic retinal injury, isolated RGC culture, elevated-pressure culture systems, or non-retinal neural tissue rather than chronic glaucomatous neurodegeneration. Consequently, the four evidence domains assessed in this review do not currently provide a basis for clinical decision-making. Furthermore, an important translational gap is the relationship between posterior segment gabapentinoid exposure and retinal neuroprotective efficacy. Although Ali et al. and Xu et al. demonstrated neuroprotective effects following oral and IP PRG administration, respectively, neither study quantified retinal drug concentrations associated with these outcomes [38,39]. Conversely, Maria et al. and Ibrahim et al. demonstrated measurable PRG concentrations in posterior segment tissues, including the retina and vitreous humor, following repeated topical ME administration in rabbits [20,21]. Maria et al. additionally demonstrated optic nerve exposure and reported significantly increased retinal, vitreous, and optic nerve PRG bioavailability with ME compared with aqueous PRG following a single topical dose [21]. However, neither study evaluated retinal neuroprotection following topical administration. While Xu et al. additionally reported improved RGC viability following direct PRG exposure at 50–200 µM in vitro, these concentrations cannot be directly equated with the total retinal tissue concentrations measured by Maria et al. because of differences in experimental model, species, exposure duration, and measurement methodology [20,21,39]. Thus, posterior segment delivery has been demonstrated, but whether topical PRG achieves sufficient exposure for retinal neuroprotection remains unresolved. Simultaneous IOP lowering and retinal neuroprotection through α2δ modulation is not implied by this review and remains a potential objective for future investigation, particularly with topical PRG formulations.

An additional consideration is ocular safety. It is important to note, however, that the human safety observations discussed below derive exclusively from systemic gabapentinoid exposure. The majority of the pharmacologic IOP studies included in this review employed topical ocular administration, with ME formulations being specifically designed to reduce systemic exposure. With this context in mind, clinical observations related to systemic PRG exposure have been heterogeneous. In an optical coherence tomography (OCT)-based study of patients with fibromyalgia (FM), longer-term PRG exposure was reported in association with thinner retinal nerve fiber layer (RNFL) measurements [48]. Interpretation of these findings is limited by evidence presented in an earlier study that FM itself has been associated with RNFL thinning independent of medication exposure [49]. In a separate OCT analysis of individuals with a history of oral PRG abuse (mean dose ~1100 mg/day) but no history of FM, no significant differences in RNFL, GCL-IPL, or GCC thickness were observed compared with controls. Within the PRG abuse group, longer duration of use was associated with modest increases in thickness in select retinal subfields, although these region-specific associations explained a limited proportion of variance and did not translate into global structural differences between groups [50]. These PRG-exposed cohorts differ substantially in population, dosing, and comorbidity profile, and their divergent findings illustrate the difficulty of isolating a drug-specific retinal structural effect from confounding population-level factors in the available human literature. Additional case reports describing reversible serous retinal or macular detachment, including central serous chorioretinopathy, during PRG exposure in both therapeutic and overdose settings further expand the spectrum of reported ocular findings. These events appear to involve choroidal and subretinal fluid dynamics rather than the neuronal layers assessed by OCT and have been observed to resolve following drug discontinuation [51,52]. A separate case report identified electroretinographic abnormalities involving rod- and cone-mediated responses despite unremarkable fundus examination and OCT findings in a patient receiving oral PRG (50 mg/day), with substantial but incomplete recovery following discontinuation [53]. Regarding anterior segment safety, a nested case–control study suggested a possible association between prior GBP exposure and incident acute angle-closure glaucoma, whereas no statistically significant association was observed for PRG in the same analysis [54]. These reports on human safety evidence are largely observational and derived from individual case reports or small, clinically heterogeneous cohorts. The absence of appropriate controls in case reports, potential confounding by underlying disease and concomitant medications, and variability in exposure duration and dosage limit causal attribution of retinal abnormalities to gabapentinoids. Similarly, associations identified through retrospective analyses cannot independently establish a direct drug-mediated mechanism. These limitations are particularly relevant when extrapolating findings from systemic administration to topical ocular formulations, for which the pharmacokinetic exposure profile differs substantially. Together, these findings do not establish a consistent pattern of ocular toxicity but highlight the need for controlled clinical evaluation and careful interpretation of heterogeneous data.

Future studies should prioritize (1) independent preclinical replication of the anterior pharmacologic findings by unaffiliated research groups, (2) longitudinal assessment in glaucomatous neurodegeneration models, (3) mechanistic clarification and integrated assessment of retinal PRG exposure and neuroprotective outcomes following topical administration, and (4) long-term safety investigations specific to topical ocular administration.

### 4.6. Limitations

Several limitations should be considered. The evidence base is almost entirely preclinical, with only one human study (a genetic association analysis) and no clinical trial data. The limited human evidence available is additionally subject to the usual constraints of observational design. Substantial heterogeneity exists across model systems, interventions, and outcome definitions: anterior segment studies largely relied on spontaneous ocular hypertension models rather than chronic glaucoma models, and posterior segment studies used acute ischemic, metabolic, or excitotoxic injury paradigms rather than chronic glaucomatous neurodegeneration. The absence of longitudinal data from established chronic glaucomatous neurodegeneration models across the entire evidence base represents the single largest gap in the current literature. No included study demonstrated both sustained IOP lowering and retinal neuroprotection within the same compound, model, or chronic disease setting. One posterior segment study also relied in part on non-retinal hippocampal slice preparations, which is mechanistically informative but does not directly reflect ocular biology [40]. This heterogeneity, together with inconsistent outcome measures and statistical reporting across studies, precluded meta-analysis and necessitated narrative synthesis, limiting pooled effect estimation. Compound-specific differences among PRG, GBP, and GBP-L further limit mechanistic interpretations and prevent the assumption that outcomes can be generalized across this drug class and its derivatives [40,41].

RoB limitations were study-specific rather than attributable uniformly to absent randomization or blinding. The neuroprotection and mechanistic evidence bodies were composed entirely of Tier II study arms, whereas the anterior segment bodies contained mostly Tier II evidence aside from two Tier I study arms within the genetic evidence (Table S6). As noted within CoE assessments, all topical PRG IOP studies originated from the same UTHSC investigative group, two members of which hold intellectual-property or commercial interests related to the evaluated formulations. Additionally, the exclusion of financially interested authors from RoB and CoE assessments reduces direct conflicts but does not eliminate potential allegiance bias arising from shared UTHSC institutional affiliations of the other authors. The internally consistent findings across mouse and rabbit experiments therefore should not be interpreted as demonstrating external reproducibility.

Finally, this review’s scope was shaped by a shared literature-screening process conducted alongside a separate review of calcium channel blockers, which may have influenced study categorization at the boundary between the two reviews. Table S3 is provided to transparently document the boundary between the two reviews by listing all publications assessed at full-text screening stage of this review and their final inclusion and exclusion decisions.

These limitations highlight the need for cautious interpretation of the available evidence and underscore the importance of future studies incorporating standardized methodologies, chronic disease models, and clinical validation to better define the role of α2δ-targeting strategies in glaucoma.

## 5. Conclusions

This systematic review synthesizes a heterogeneous, predominantly preclinical evidence base of α2δ-associated biology across anterior segment IOP regulation, retinal neuroprotection, and mechanistic signaling relevant to glaucoma pathophysiology. In anterior segment models, pharmacologic targeting of α2δ1 with PRG was consistently associated with reductions in IOP, including a dose–response relationship, formulation-dependent prolongation of effect, and maintained response during repeated once-daily dosing. Certainty was moderate within this preclinical pharmacologic evidence domain, but all intervention studies originated from a single investigative group. Separately, genetic evidence linking *CACNA2D1*/*Cacna2d1* to IOP-related phenotypes and POAG susceptibility was rated as moderate certainty and provides target-level biological context. Neuroprotective evidence was rated as low certainty and was derived from diabetic retinal injury, acute retinal ischemia/reperfusion, and isolated RGC models rather than chronic glaucomatous neurodegeneration. Mechanistic evidence was likewise rated as low certainty and included distinct experimental findings involving TSP2-α2δ1-associated retinal signaling and ischemia-related neurotransmitter release. These evidence bodies differ substantially in compound, tissue, species, model, and endpoint and should not be interpreted as demonstrating a unified therapeutic mechanism. The possibility that α2δ-associated pathways may influence both anterior and posterior segment processes remains a hypothesis for future testing rather than an established mechanism or validated dual-action therapeutic strategy. Independent pharmacologic replication, evaluation in chronic glaucomatous neurodegeneration models, and long-term topical safety investigations are needed before clinical relevance can be suggested.

## Figures and Tables

**Table 1:** Characteristics of included studies. Full study characteristics and quantitative outcomes are available in Tables S4 and S5, respectively.

Study	Domain	Model	Intervention	Comparator	Outcomes	Main Findings
Chintalapudi 2017 [19]	IOP	C57BL/6J (B6) mouse in vivo; BXD mouse in vivo; DB rabbit in vivo; human GWAS	Genetic variation; topical PRG solution (0.3–1.2%)	Vehicle; allelic variation; controls	IOP; POAG risk	*Cacna2d1* regulates IOP in BXD mice; PRG lowers IOP dose-dependently in B6 mice; 0.9% PRG lowers IOP in BXD mice and DB rabbits; human *CACNA2D1* variants associated with POAG
Lankford 2024 [29]	IOP	Mouse in vivo gene KO; human TM cells	*Cacna2d1* KO; PRG solution 10 and 100 μM	WT mice; untreated human TM cells	IOP; TM signaling	*Cacna2d1* KO modestly reduces IOP in mice; PRG does not alter human TM contractility pathways in short term culture
Ibrahim 2019 [20]	IOP	DB rabbit in vivo	Topical 0.6% PRG ME (single daily dose, 21-day chronic dosing)	Different formulations of 0.6% PRG ME; 0.6% PRG solution; vehicle	IOP; AUC; safety	Single-dose PRG ME enhances magnitude and duration with sustained efficacy; 21-day dosing reveals excellent ocular safety, maintained efficacy, and high drug deposition in different ocular tissues.
Maria 2024 [21]	IOP	DB rabbit in vivo; C57BL/6J (B6) mouse in vivo, BXD mouse in vivo	Single-dose topical PRG ME (0.1–0.7%); 60-day chronic dosing topical PRG ME (0.6%)	Vehicle; latanoprost	IOP; AUC; duration; safety; lack of tachyphylaxis	PRG ME demonstrates increased duration and AUC in B6 and BXD mice; PRG ME has comparable peak effect to latanoprost with prolonged duration in DB rabbits; PRG ME displays excellent safety profile and no tachyphylaxis after 60 days of once-daily dosing; ME formulation increased PRG exposure in posterior segment tissues after a single-dose; similarly increased exposure was also demonstrated after 60 days of once-daily dosing.
Li 2021 [37]	IOP	DB rabbit in vivo	Topical 0.6% solutions of PRG, acivicin, or zoledronic acid	Vehicle	IOP	PRG solution produces greatest IOP reduction among tested agents
Ali 2019 [38]	Neuro-protection	Rat in vivo (DR)	PRG (3, 10, 30 mg/kg oral)	Diabetic control	Retinal thickness; GCL cells; inflammation	PRG reduces inflammation and preserves retinal structure and cellularity
Xu 2022 [39]	Neuro-protection	C57BL/6J (B6) mouse in vivo (RIR)	PRG (15–60 mg/kg IP)	Vehicle	Retinal thickness; RGC survival	PRG preserves retinal structure and reduces apoptosis
Jehle 2000 [40]	Neuro-protection; Mechanistic	Rat in vivo (retinal ischemia); Rat in vitro hippocampal slice	GBP-L; GBP (50 mg/kg IP; 100 µM superfusion baths)	Vehicle	RGC survival; Hippocampal glutamate release	GBP-L improves RGC survival in vivo. GBP-L also reduces glutamate release in a hippocampal slice preparation (non-retinal in vitro model); GBP is less effective
Pielen 2004 [41]	Neuro-protection	Rat in vitro RGC culture	GBP-L 3.2–320 µM; GBP 32 µM	Control	RGC survival	GBP-L enhances RGC survival via proposed KATP-dependent mechanism
Wang 2017 [15]	Mechanistic	Rat in vitro retinal neuron-macroglia culture	Recombinant TSP2; GBP 32 µM	Control	Synaptic proteins	TSP2 drives α2δ1-dependent synaptic remodeling that is inhibited by GBP
Hu 2022 [16]	Mechanistic	Rat in vivo (RIR)	GBP 100 mg/kg; TSP2 knockdown	Control	Axonal integrity; synapses	GBP disrupting TSP2-α2δ1 signaling reduces injury-induced remodeling

DB: Dutch belted; GWAS: Genome-Wide Association Study; POAG: primary open-angle glaucoma; KO: knockout; ME: microemulsion; AUC: area under the curve; DR: diabetic retinopathy; GCL: ganglion cell layer; RIR: retinal ischemia–reperfusion; IP: intraperitoneal; GBP: gabapentin; GBP-L: gabapentin-lactam; KATP: ATP-sensitive potassium channel.

## Data Availability

No new primary experimental data were generated in this study. Secondary materials generated during the review process, including the search strategy (Table S2), study characteristics and quantitative outcomes (Tables S4 and S5), Risk of Bias assessments (Table S6), and the Certainty of Evidence assessment (Table S7), are available without restriction.

## Supplementary Materials

The following supporting information can be downloaded at https://www.mdpi.com/article/10.3390/jcm15218681/s1: PRISMA 2020 checklist (Table S1), search strategy (Table S2), full text screening details (Table S3), study characteristics and quantitative outcomes (Table S4 and Table S5), Risk of Bias assessments (Table S6), and the Certainty of Evidence assessment (Table S7).
