# Supplementary material for: Pregabalin and α2δ Subunit Modulation: A Systematic Review of Preclinical Evidence for Intraocular Pressure Regulation and Retinal Neuroprotection
Source: J Clin Med. 2026 Nov 6;15(21):8681. doi: 10.3390/jcm15218681 (PMC13764461; doi:10.3390/jcm15218681)
Supplement: Supplementary file 1 [file jcm-15-08681-s001.zip › Table S2 (Search Strategies).pdf]

**PubMed (348 results)**	(glaucoma[MeSH] OR glaucoma[tiab] OR "ocular hypertension"[tiab] OR "intraocular pressure"[tiab] OR IOP[tiab] OR "optic nerve"[tiab] OR "retinal nerve fiber layer"[tiab] OR RNFL[tiab] OR "visual field"[tiab] OR "visual fields"[tiab] OR "retinal ganglion cell layer"[tiab] OR RGC[tiab]) AND (gabapentin[MeSH] OR gabapentin[tiab] OR pregabalin[MeSH] OR pregabalin[tiab] OR gabapentinoid*[tiab] OR "calcium channel blocker"[tiab] OR "calcium channel blockers"[tiab] OR dihydropyridine*[tiab] OR amlodipine[tiab] OR nifedipine[tiab] OR felodipine[tiab] OR verapamil[MeSH] OR verapamil[tiab] OR diltiazem[MeSH] OR diltiazem[tiab] OR "non-dihydropyridine"[tiab] OR CACNA2D*[tiab] OR alpha2delta*[tiab] OR "alpha-2-delta-1"[tiab] OR "alpha-2-delta-2"[tiab] OR "alpha2delta-1"[tiab] OR "alpha2delta-2"[tiab] OR α2δ*[tiab] OR "α2δ-1"[tiab] OR "α2δ-2"[tiab] OR "alpha2delta"[tiab]) AND hasabstract
**Scopus (1,111 results)**	TITLE-ABS-KEY(glaucoma OR glaucoma* OR "ocular hypertension" OR "intraocular pressure" OR IOP OR "optic nerve" OR "retinal nerve fiber layer" OR RNFL OR "visual field" OR "retinal ganglion cell layer" OR RGC) AND TITLE-ABS-KEY((CACNA2D* OR alpha2delta* OR "alpha-2-delta" OR "alpha-2-delta-1" OR "alpha-2-delta-2" OR "alpha2delta-1" OR "alpha2delta-2" OR "calcium channel subunit") OR (gabapentin OR pregabalin OR gabapentinoid* OR dihydropyridine OR dihydropyridines OR amlodipine OR nifedipine OR felodipine OR "non-dihydropyridine" OR verapamil OR diltiazem)) AND DOCTYPE(ar) AND NOT DOCTYPE(cp)
**Cochrane (89 results)**	(glaucoma* OR "ocular hypertension" OR "intraocular pressure" OR IOP OR "optic nerve" OR "retinal nerve fiber layer" OR RNFL OR "visual field" OR "visual fields" OR "retinal ganglion cell layer" OR RGC) AND ((gabapentin OR pregabalin OR gabapentinoid* OR "calcium channel blocker" OR "calcium channel blockers" OR dihydropyridine OR dihydropyridines OR amlodipine OR nifedipine OR felodipine OR verapamil OR diltiazem OR "non-dihydropyridine") OR (CACNA2D* OR alpha2delta* OR "alpha-2-delta" OR α2δ* OR "calcium channel subunit" OR "calcium channel subunits"))
**Embase (1,146 results)**	(glaucoma/exp OR 'ocular hypertension'/exp OR 'intraocular pressure'/exp OR glaucoma:ab,ti OR 'ocular hypertension':ab,ti OR 'intraocular pressure':ab,ti OR IOP:ab,ti OR 'optic nerve'/exp OR 'optic nerve':ab,ti OR 'retinal nerve fiber layer'/exp OR 'retinal nerve fiber layer':ab,ti OR RNFL:ab,ti OR 'visual field'/exp OR 'visual field':ab,ti OR 'visual fields':ab,ti OR 'retinal ganglion cell layer'/exp OR 'retinal ganglion cell layer':ab,ti OR RGC:ab,ti) AND (gabapentin/exp OR gabapentin:ab,ti OR pregabalin/exp OR pregabalin:ab,ti OR gabapentinoid*:ab,ti OR 'calcium channel blocker'/exp OR 'calcium channel blocker':ab,ti OR 'calcium channel blockers':ab,ti OR dihydropyridine derivative/exp OR dihydropyridine*:ab,ti OR amlodipine:ab,ti OR nifedipine:ab,ti OR felodipine:ab,ti OR verapamil/exp OR verapamil:ab,ti OR diltiazem/exp OR diltiazem:ab,ti OR 'non-dihydropyridine':ab,ti OR CACNA2D*:ab,ti OR alpha2delta*:ab,ti OR 'alpha-2-delta-1':ab,ti OR 'alpha-2-delta-2':ab,ti OR 'alpha2delta-1':ab,ti OR 'alpha2delta-2':ab,ti OR α2δ*:ab,ti OR 'α2δ-1':ab,ti OR 'α2δ-2':ab,ti OR alpha2delta:ab,ti) NOT (conference abstract OR conference paper OR letter OR note OR editorial)
