# Supplementary material for: Pregabalin and α2δ Subunit Modulation: A Systematic Review of Preclinical Evidence for Intraocular Pressure Regulation and Retinal Neuroprotection
Source: J Clin Med. 2026 Nov 6;15(21):8681. doi: 10.3390/jcm15218681 (PMC13764461; doi:10.3390/jcm15218681)
Supplement: Supplementary file 1 [file jcm-15-08681-s001.zip › Table S7 (Certainty of Evidence Assessments).pdf]

Table S7. GRADE/OHAT assessment of certainty of evidence by outcome.

Outcome	Included Studies	Study Designs	Initial Confidence Rating	Factors Decreasing Confidence	Factors Increasing Confidence	Overall Certainty
**Anterior Segment (pharmacology)**	5 studies, 5 arms [19-21,29,37]	In vivo animal (4 study arms); in vitro human (1 study arm)	High (++++)	Risk of Bias (-)<br>Indirectness (-)<br>Publication bias (-)	Consistency across models and species (+)<br>Dose response gradient (+)	Moderate (+++)
**Anterior Segment (genetics)**	2 studies, 3 arms [19,29]	In vivo animal (2 study arms); human genetic association (1 study arm)	High (++++)	Indirectness (-)<br>Publication bias (-)	Consistency across models and species (+)	Moderate (+++)
**Neuroprotection**	4 studies, 4 arms [38-41]	In vivo animal (3 study arms); in vitro animal (1 study arm)	High (++++)	Risk of bias (-)<br>Indirectness (-)	None	Low (++)
**Mechanistic**	3 studies, 3 arms [15,16,40]	In vivo animal (1 study arm); in vitro animal (2 study arms)	High (++++)	Risk of bias (-)<br>Indirectness (-)	None	Low (++)

GRADE: Grading of Recommendations, Assessment, Development and Evaluations; OHAT: Office of Health Assessment and Translation; IOP: intraocular pressure.

Certainty of evidence (CoE) was assessed at the level of four prespecified outcome bodies of evidence (anterior segment pharmacology, anterior segment genetics, neuroprotection, and mechanistic) using the GRADE/OHAT framework. Initial confidence was assigned according to the key design features specified by OHAT, and subsequent downgrading or upgrading was based on the characteristics of the body of evidence rather than on any single study. Risk of Bias (RoB) judgments were drawn directly from the study-arm-level assessments in Table S6. Consistent with OHAT guidance, an evidence body-level RoB downgrade was reserved for bodies of evidence in which methodological limitations were sufficiently prevalent or consequential across the contributing studies to raise meaningful doubt about the findings, rather than being applied automatically because individual studies contained one or more "probably high" risk items.

All five study arms of the anterior segment pharmacology body satisfied the four OHAT key design features required for high initial confidence (controlled exposure, correct temporal sequence, individual-level data, and comparison group). All five study arms were classified as Tier II studies (Table S6) per the OHAT RoB tool. Limitations across the body evidence included randomization, allocation concealment, and investigator blinding, while study-specific limitations included incomplete reporting of variance assumptions, possible contralateral exposure or treatment-order effects in some paired-eye experiments, incomplete exposure characterization in Maria et al., and uncertainty regarding biological replication and control conditions in the Lankford et al. TM cell experiments. With all studies being rated as Tier II, RoB concerns across the anterior pharmacology body of evidence were sufficient to warrant an evidence body-level RoB downgrade. Confidence was also downgraded for indirectness, because the in vivo evidence was obtained predominantly in non-glaucomatous experimental animals and the topical formulations have not yet been evaluated in published clinical trials. Confidence was further downgraded for risk of publication bias given the small sample sizes and the concentration of the literature within a single investigator group. The null TM cell finding from Lankford et al. was explicitly considered in this assessment. Because it addresses a distinct mechanistic endpoint (TM cytoskeletal/contractility signaling) rather than the IOP-lowering outcome itself, and because section 4.2 of the manuscript interprets it as arguing against one specific proposed mechanism rather than contradicting the overall pharmacodynamic IOP data, it was not treated as introducing unexplained inconsistency into the body of evidence. Confidence of the anterior pharmacology body of evidence was upgraded once for cross-species consistency, as findings were replicated across mice

and rabbits. Confidence was further upgraded for evidence of a dose response relationship, as Chintalapudi et al. and Maria et al. both demonstrated an overall monotonic dose response gradient for topical PRG in both mice and rabbits, with saturation at higher concentrations (0.9% for topical PRG solution in mice, 0.6% for PRG ME in rabbits). After accounting for these downgrades and upgrades, the overall certainty of the anterior segment pharmacology evidence was rated as moderate. This rating reflects confidence in the reported preclinical IOP-lowering response rather than a suggestion of clinical efficacy or an assumption of independent reproducibility.

The anterior segment genetics body of evidence comprised the human GWAS and mouse systems-genetics arms of Chintalapudi et al. and the α2δ1-null mouse study of Lankford et al. These studies also began at high initial confidence per the OHAT criteria described above. Because genotypes are inherited rather than experimentally assigned, randomization and allocation-concealment domains were considered not applicable to the mouse genetic studies. Potential group comparability and residual confounding sources of bias were instead evaluated under the applicable experimental-condition and other-bias domains in Table S6. The human GWAS and mouse-system genetics arms of Chintalapudi et al. were both classified as Tier I, whereas the Lankford et al. mouse genetic arm was classified as Tier II. The principal concerns included incomplete control of the age distribution in BXD mouse analyses, despite an age-dependent *Cacna2d1*-IOP association, and incomplete reporting of attrition, within-litter clustering, and potential developmental or homeostatic compensation in the constitutive α2δ1 knockout model. These limitations were not considered sufficiently uniform or severe to justify an evidence body-level RoB downgrade. Confidence was downgraded for indirectness and risk of publication bias because the evidence base remains small and primarily establishes genetic association or target-level effects rather than clinical glaucoma treatment effects. Confidence was upgraded for consistency of the relationship between α2δ1/*Cacna2d1*/*CACNA2D1* biology and incidence of both ocular hypertension and primary open-angle glaucoma across genetic analyses. The resulting overall certainty was moderate.

Each of the study arms of the neuroprotection body of evidence met the OHAT design criteria for high initial confidence as described above, but all four were classified as Tier II in Table S6, supporting an evidence body downgrade for RoB. The underlying limitations differed between studies rather than representing one uniform methodological defect (refer to Table S6 for full detailed rationale behind the assessments of these limitations). Thus, the RoB downgrade reflects the cumulative pattern of study-specific limitations across a uniformly Tier II evidence base. CoE was additionally downgraded for indirectness because the studies used heterogenous models of acute retinal injury, resulting in an evidence base that overall cannot be assumed to represent pathological changes of chronic glaucomatous neurodegeneration. Differences between GBP and GBP-L in the Jehle et al. in vivo arm were considered biologically explainable by the distinct KATP-channel-dependent activity attributed to GBP-L rather than unexplained inconsistency. No upgrading factor was applied, yielding low overall certainty for neuroprotective evidence.

The three arms of the mechanistic body of evidence all met the OHAT design features for high initial confidence but were classified as Tier II in Table S6, resulting in a RoB downgrade. In adapting the OHAT animal-study criteria to in vitro experiments, absent allocation concealment or personnel blinding was not considered intrinsically high risk where treatment was applied to standardized cell or tissue preparations and investigators had little practical opportunity to alter the biological outcome through knowledge of treatment assignment. Instead, the downgrade was driven by more consequential study-specific issues similar to the neuroprotection body of evidence. CoE was also downgraded for indirectness because these experiments relied on isolated retinal cultures, elevated hydrostatic-pressure systems, or, in Jehle et al., non-ocular hippocampal tissue rather than intact chronic ocular hypertension models. The three studies were considered mechanistically complementary rather than sufficiently independent and comparable to justify an upgrade for consistency. This yielded low overall certainty for mechanistic evidence.
